# Convergent evolution of complex genomic rearrangements in two fungal meiotic drive elements

**DOI:** 10.1038/s41467-018-06562-x

**Published:** 2018-10-12

**Authors:** Jesper Svedberg, Sara Hosseini, Jun Chen, Aaron A. Vogan, Iva Mozgova, Lars Hennig, Pennapa Manitchotpisit, Anna Abusharekh, Thomas M. Hammond, Martin Lascoux, Hanna Johannesson

**Affiliations:** 1 Department of Organismal Biology, Uppsala University, Norbyvägen 18D, 752 36 Uppsala, Sweden; 2 Department of Ecology and Genetics, Science for Life Laboratory, Uppsala University, Norbyvägen 18D, 752 36 Uppsala, Sweden; 3 Department of Plant Biology and Linnean Center for Plant Biology, Swedish University of Agricultural Sciences, PO-Box 7080, SE-75007 Uppsala, Sweden; 4 School of Biological Sciences, Illinois State University, Normal, IL 61790 USA; 5 Present Address: Institute of Microbiology of the Czech Academy of Sciences, Centre Algatech, Opatovický mlýn, CZ-37981 Třeboň, Czech Republic

## Abstract

Meiotic drive is widespread in nature. The conflict it generates is expected to be an important motor for evolutionary change and innovation. In this study, we investigated the genomic consequences of two large multi-gene meiotic drive elements, *Sk-2* and *Sk-3*, found in the filamentous ascomycete *Neurospora intermedia*. Using long-read sequencing, we generated the first complete and well-annotated genome assemblies of large, highly diverged, non-recombining regions associated with meiotic drive elements. Phylogenetic analysis shows that, even though *Sk-2* and *Sk-3* are located in the same chromosomal region, they do not form sister clades, suggesting independent origins or at least a long evolutionary separation. We conclude that they have in a convergent manner accumulated similar patterns of tandem inversions and dense repeat clusters, presumably in response to similar needs to create linkage between genes causing drive and resistance.

## Abstract (web summary)

Meiotic drive elements are selfish genetic elements that mediate a skew in their sexual transmission from parent to offspring. Here, the authors sequence and analyze large and complex genomic regions associated with meiotic drive elements in the fungus *Neurospora intermedia*.

## Introduction

A significant portion of eukaryote genomes consists of selfish or parasitic genetic elements. Such elements have a transmission advantage relative to other parts of the genome but are either neutral or detrimental to the fitness of the organism as a whole^1,2^. Despite such genomic conflicts being known since the early days of modern genetics, only recently has there been a clear awareness that genetic conflicts could be important engines for evolutionary change^2,3^. Meiotic drive elements (MDEs) constitute a class of selfish genetic elements that are located in the nuclear genome and mediate a skew in their own sexual transmission from parent to offspring, causing them to be recovered in more than half of the surviving meiotic products^1,4^. This phenomenon is referred to as meiotic drive, and with even minimal skews in transmission frequency, it can lead to quick fixation or stable polymorphisms of a driving allele^5,6^. Many MDEs require the interaction of multiple genes or genetic components to function, which is expected to lead to strong selection for linkage, especially in systems where unlinking distinct loci that confer killing and resistance would lead to self-killing. Accordingly, meiotic drivers are often associated with structural rearrangements that reduce recombination or are positioned in regions of low recombination, such as centromeres, heterochromatic regions, or non-recombining sex chromosomes^1,5^. This is the case for several of the most well-studied meiotic drive systems, including *Segregation Distorter* (SD) complex in *Drosophila melanogaster*^7^, the *t haplotype*^8^ in mice, and several so-called selfish sex chromosomes in different species of animals and plants^9^.

A multilocus MDE can be considered a supergene, the term used to refer to a group of functionally related genes that are linked together and, hence, segregate as a single unit^10,11^. In many supergenes, for instance sex chromosomes and fungal mating-type chromosomes, the reduced recombination has resulted in rapid divergence between the non-recombining haplotypes and a reduced ability to purge deleterious mutations through recombination^12–16^. This is also the case for many MDEs, which have accumulated recessive lethal mutations^17^, rearrangements such as segmental duplications and deletions^8^, and show an expansion of repetitive regions^18^. Furthermore, patterns of divergence between MDEs and non-driving individuals have been studied in several species^8,19,20^, inversions have been observed in cytological examinations^17,20^, and nucleotide polymorphism has been studied using markers and whole-genome single-nucleotide polymorphism (SNP) data^8,19,21^. However, the full extent of divergence can be challenging to study even in model organisms with high-quality genomic resources because discovery and mapping of complex rearrangements typically requires de novo genome assembly of the complete MDE.

In fungi, meiotic drive manifests itself as spore killing, where sexual spores carrying a spore killer genotype destroy spores that carry a sensitive genotype. Spore killing has so far only been observed in the ascomycete fungi, and several different killer systems are identified in *Podospora anserina*^22,23^, *Schizosaccharomyces pombe*^24,25^, and two species of *Neurospora*^26^. While the spore killers in *P. anserina* and *S. pombe* are single-gene systems, *Spore killer-2* (*Sk-2*) and *Spore killer-3* (*Sk-3*) are complex MDEs found in *N. intermedia* that map to a 30 cM region surrounding the centromere on chromosome 3 where recombination is suppressed between killer and sensitive strains^27^. A region necessary for killing (*rfk-1*) has been mapped in *Sk-2* to the right edge of the region of suppressed recombination^28,29^ and a gene that confers resistance against both *Sk-2-* and *Sk-3*-based killing (*rsk*) has been identified at the left flank^30^. Depending on the allelic variant of the resistance gene a strain carries, it shows either resistance to *Sk-2* or *Sk-3* or sensitivity to killing^30^. The cause of the suppression of recombination is not known, but an inversion covering at least 200 kb was previously identified near the *rfk-1* region in one *Sk-2* strain^28^. Furthermore, the mechanism of killing in the *Sk-2* and *Sk-3* systems has not been elucidated, but Hammond et al.^30^ proposed a toxin–antitoxin model, in which the killer gene encodes a toxin that can kill a spore, and the resistance gene encodes an antitoxin that ensures the survival of the spore that produces it. Consequently, when two strains of the same spore killer are crossed, all spores will survive, while crosses of *Sk-2* and *Sk-3* result in the production of empty asci^26^, which has been interpreted as mutual killing by incompatible toxin–antitoxin systems^30^.

The purpose of this study was to investigate the consequences of multilocus meiotic drive on genome architecture in *N. intermedia*. We generated near-complete genome assemblies using PacBio long-read sequencing data from all five available spore killer strains (four *Sk-2* and one *Sk-3*) and one sensitive strain of *N. intermedia* and assembled the complete non-recombining region in all of these. We also produced a detailed annotation of gene content, repeat content, and distribution of heterochromatin and euchromatin from one strain of each killer type and the sensitive strain. Short-read Illumina sequencing was used for phylogenetic and population genetic analysis of a large collection of *N. intermedia* strains from natural populations. Using this large and diverse dataset, we show that even though *Sk-2* and *Sk-3* are located in the same chromosomal region they do not cluster together in phylogenetic analyses, suggesting separate origins. Both the *Sk-2* and *Sk-3* haplotypes have accumulated a dense set of inversions that are interspersed with transposable elements (TEs). The inversions are unique for each killer type, further supporting a model with an ancient split. In the non-recombining region of *Sk-2*, we identified signs of relaxed selection, in agreement with the hypothesis that recombination suppression reduces the efficacy of selection in this region. For example, TEs have spread in the non-recombining regions of both *Sk-2* and *Sk-3* despite what appears to be a set of fully functional mechanisms to limit their spread.

In conclusion, our data show that at least two complex MDEs have established themselves in *N. intermedia* and, during this process, have induced independent and convergent structural changes in the same genomic region.

## Results

### Killer-specific inversions are found in the *Sk* regions

*Neurospora intermedia* is a close relative to the model species *N. crassa*, from which it diverged 3–4 million years ago (MYA), and it diverged from its closest relative *N. metzenbergii* around 2 MYA^31^. Spore killing was discovered in a small number of natural isolates of *N. intermedia* in south-east Asia in the 1970s, and most isolates were introgressed into different genetic backgrounds for increased tractability in the laboratory^26^. Today, only one natural isolate of *Sk-2* is available at culture collections, while the other three *Sk-2* isolates and the single *Sk-3* isolate are available only as backcrosses to *N. intermedia* and *N. crassa* laboratory strains.

**Fig. 1:**
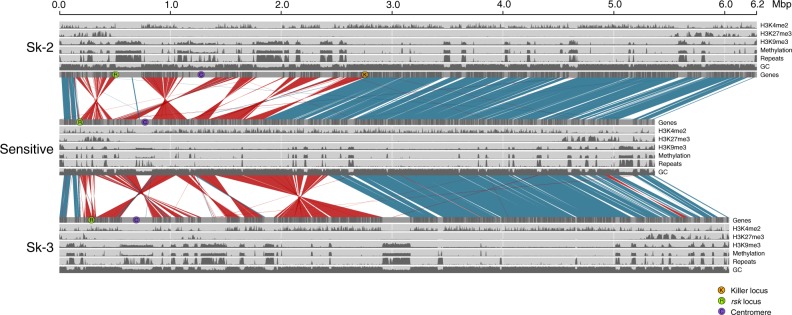
An alignment of chromosome 3 in *Sk-2* *and Sk-3* to sensitive *Neurospora intermedia*, which retains the ancestral gene order, shows a series of large inversions in the region between the killer and resistance loci. Blue color shows collinear regions and red inverted regions. All inversions are different between *Sk-2* and *Sk-3* and are interspersed with gene-poor and repeat-rich inserted regions. The location of the *Sk-2* killer locus us marked with an orange circle and the *rsk* (resistance) locus and the centromeres are marked with green and purple circles, respectively. Tracks in gray show genes; GC content; repeat content; cytosine methylation; and H3K9me3, H3K27me3, and H3K4me2 histone methylation. H2K9me3 is a mark for constitutive heterochromatin, which in *Neurospora* is highly associated with repeats; H3K27me3 is a mark for facultative heterochromatin, which is primarily found in subtelomeric regions; and H3K4me2 is a mark for euchromatic regions. Cytosine methylation and the three different histone modifications were estimated using three biological replicates, and here the sum of the triplicates are presented

The analysis of the genomes in this study (Supplementary Table 1), including all currently available spore killer strains of *N. intermedia*, show that the chromosomal region containing *Sk-2* and *Sk-3* in *N. intermedia* represents an exception to the high degree of collinearity previously reported in *Neurospora*^32^. Specifically, we used the PacBio RSII platform to generate six high-quality genome assemblies with full or nearly full chromosome-length contigs (Table 1, Supplementary Fig. 1). In order to identify structural rearrangements, we aligned these assemblies against the *N. crassa* OR74 reference genome^33^ and against each other. These alignments show that the sensitive *N. intermedia* strain is collinear with *N. crassa* (as was previously shown for a different sensitive strain^32^), but the *Sk-2* and *Sk-3* strains carry clusters of large inversions on chromosome 3 in the region where recombination between killer and sensitive strains has previously been reported to be suppressed^27^ (Fig. 1, Supplementary Table 2). For both *Sk-2* and *Sk-3*, the inversions are non-overlapping and situated next to each other, without any regions retaining the ancestral gene order interspersed between them (Fig. 1, Supplementary Table 2). The four sequenced *Sk-2* genomes carry the same set of inversions in the inverted region (from here on referred to as the *Sk* region), but strain 7429 also carries an extra ~1.3 Mbp inversion on the right chromosome arm, outside of the region that showed suppressed recombination in previous studies (Supplementary Fig. 2). The *Sk-3* genome carries several tandem inversions in the same region as the *Sk-2* strains (Fig. 1), but the breakpoints of all *Sk-3* inversions differ from those in *Sk-2*, indicating that they have accumulated independently (Fig. 1, Supplementary Table 2).

**Table 1:** PacBio sequencing and genome assembly statistics for seven *N. intermedia* strains

Strain^a^	Phenotype	Location	Mean coverage	Mean subread length (bp)	Assembly size (Mbp)	Contigs	Mapped contigs^b^
7426	*Sk-2*	Sabah, Malaysia	60.5	4693	42.2	31	9
7401	*Sk-2*	Brunei	94.5	8645	42.1	23	7
7427	*Sk-2*	Java, Indonesia	84	8295	42.8	23	7
7429	*Sk-2*	Papua New Guinea	60.5	7150	41.5	31	8
3194	*Sk-3*	Papua New Guinea	77.4	5673	41.7	24	8
8761	Sensitive	Taiwan	143	6175	41.2	27	9
8807^c^	Sensitive	India	69.3	8602	41.3	14	7

^a^Strain numbers correspond to the strain ID in the Fungal Genetics Stock Center (FGSC)

^b^Number of contigs mapping to the seven chromosomes of the *N. crassa* OR74A reference genome

^c^The genome of strain 8807 was initially published in Sun et al. (2017)^32^ but was generated together with the rest of the genomes in this study

### *Sk*s include repetitive regions enriched for heterochromatin

**Fig. 2:**
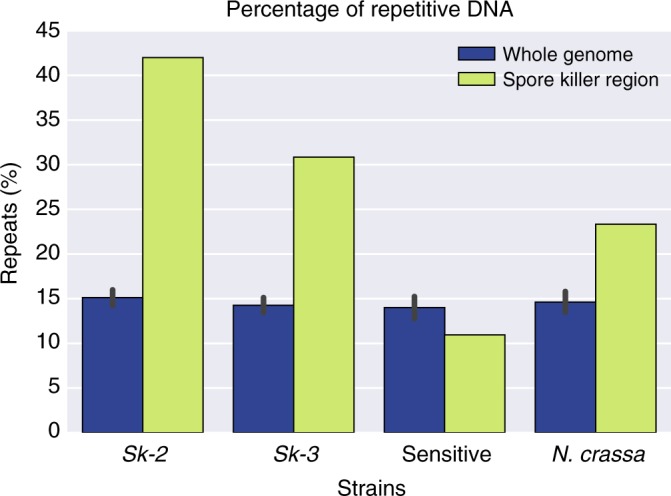
Percentage of repetitive DNA in three strains of *Neurospora intermedia* with different spore killer phenotypes (Sk-3: 3194; Sk-2: 7426; sensitive: 8761). Blue bars show the whole genome, and yellow bars the non-recombining spore killer region. Error bars (95% confidence intervals) for the whole-genome data were calculated using sliding windows of the same size as the spore killer region, with a step size of 200 kb. Note that no formal statistical test has been performed to determine if the repeat content in the spore killer region is significantly different from the background genome, but the data point does not overlap with the 95% confidence interval from the whole genome

Both *Sk-2* and *Sk-3* haplotypes are enriched for repetitive sequences (Fig. 1, Fig. 2, Table 2), which are mainly found in large, dense repeat clusters bordering the inverted regions (Fig. 1). The repeats are primarily the remains of TEs. Several TE families appear to have expanded preferentially (Supplementary Tables 3 and 4), although these correspond to no more than 20% of the repetitive content in the region (Table 2, Supplementary Table 4). TEs are enriched in the *Sk* haplotype both when compared to the rest of the genome and compared to the homologous region in the sensitive strains of *N. intermedia* and *N. crassa* (Fig. 2, Table 2).

**Table 2:** Repetitive content in genomic regions of *Sk-2*, *Sk-3*, and sensitive strains of *N. intermedia*

Strain	Phenotype	Whole genome	Spore killer region	Enrichment in spore killer region^a^	Fraction of enriched repeat families^b^
3194	*Sk-3*	17%	31%	1.81	4.25%
7426	*Sk-2*	18%	42%	2.36	19.4%
8761	Sensitive	16%	11%	0.67	0%
*N. crassa*	Sensitive	16%	23%	1.43	19.2%

^a^Repeat density in spore killer region compared to the genome average

^b^Fraction of repetitive content consisting of enriched repeat families (see Supplementary Table 4)

**Fig. 3:**
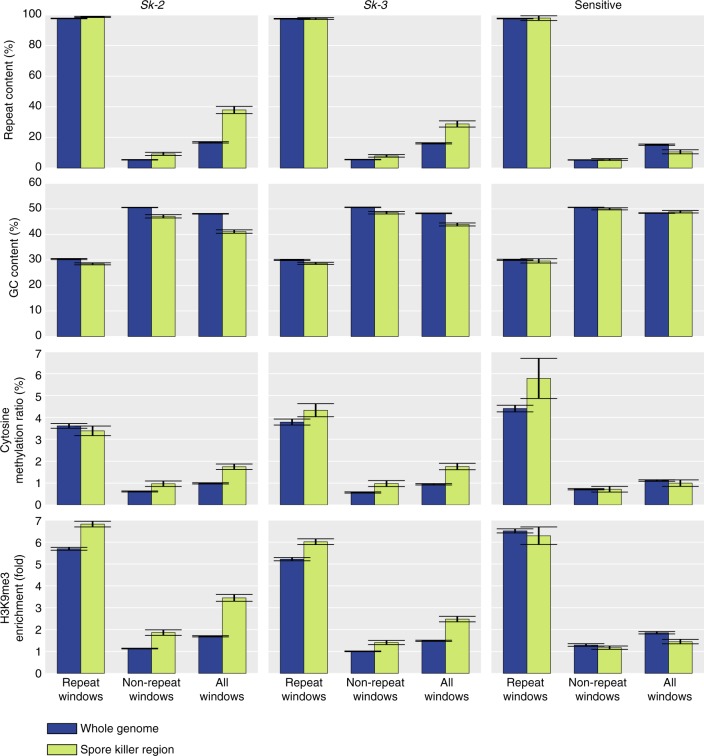
Repeat content, GC content, cytosine methylation, and H3K9me3 enrichment of the genome and the spore killer region in strains 3194 (*Sk-3*), 7426 (*Sk-2*), and 8761 (sensitive) of *Neurospora intermedia*. All values are calculated using 2 kb sliding windows with a step size of 2 kb. Windows consisting of >75% repetitive content are classified as “repeat windows” and those with <75% are classified as “non-repeat windows.” “Repeat content” plots indicate that most windows classified as repetitive contain close to 100% repeats, consistent with a clustered distribution of repeats. Error bars show 95% confidence intervals. GC content, cytosine methylation levels, and H3K9me3 levels are all significantly different between repeat and non-repeat windows (Student’s *T* test, *p* ≪ 0.001 for all comparisons). The *Sk* regions of the spore killers are also significantly different from the rest of the genome in all these characteristics, though the differences are less pronounced than when comparing repetitive and non-repetitive regions. These results are consistent with previous data from *N. crassa*, in which cytosine methylation and H3K9me3 histone methylation are both signatures of constitutive heterochromatin and primarily found in repetitive regions that have been mutated by the RIP mechanism, which also lowers the GC content

In *N. crassa*, a wide repertoire of mechanisms thought to defend against the expansion repetitive sequences are present^34^. Most notable is the Repeat-Induced Point mutation (RIP) system, which induces C-to-T mutations in any sequence appearing more than once in the genome. RIP is hypothesized to defend the genome from TEs^35^ and can inactivate these through mutations, lowers their average GC content, and also acts as a signal for heterochromatin formation^36^ and DNA methylation^37^, which both are expected to limit TE activity. Accordingly, the GC content of the repeat clusters in the *Sk* haplotypes is lower than that of genic regions (~30% vs 50%: Figs. 1 and 3). This is also the case in the whole genome of both the killer and sensitive strains, indicating that the expansion of repeats in the *Sk-2* and *Sk-3* haplotypes is not due to a less efficient RIP system in this region. In order to determine whether the heterochromatin structure is different from that found in *N. crassa* and whether a different heterochromatin structure could explain the *Sk* repeat expansions, we performed bisulfite sequencing and chromatin immunoprecipitation sequencing (ChIP-seq) of one *Sk-2*, one *Sk-3*, and one sensitive *N. intermedia* strain (Supplementary Tables 5 and 6). These analyses revealed that cytosine methylation and H3K9me3 (histone 3 lysine 9 tri-methylation) (which are both signatures of constitutive heterochromatin in *N. crassa*^36^) are enriched in repetitive regions (Figs. 1 and 3), and H3K4me2 and H3K27me3 methylation marks (signatures of euchromatin and facultative heterochromatin, respectively) are absent in the same regions^38^ (Fig. 1). In summary, we did not find a signal of reduced ability to defend against the expansion of TEs in the spore killer regions.

### Novel gene content in the *Sk* regions

To evaluate whether the *Sk* regions have accumulated novel open reading frames (ORFs), the gene content of the spore killer strains and sensitive strains was compared. We gathered transcriptome data from vegetative tissue and from crosses involving four strains: one *Sk-2*, one *Sk-3*, and two sensitives (Supplementary Tables 7 and 8), and used these data to annotate the genome assemblies. Unique genes in the *Sk-2* and *Sk-3* regions were identified by comparing our gene annotations of the spore killer strains to *N. crassa* and *N. intermedia* sensitive strains and to each other (Supplementary Table 9, Supplementary Fig. 3). We identified 6, 4, and 5 unique ORFs in the *Sk* region of *Sk-2*, *Sk-3*, and sensitive *N. intermedia*, respectively (Supplementary Table 9), but the majority of these unique genes could not be assigned a known function. We also identified three genes that are found in both *Sk-2* and *Sk-3* but not in any of the sensitive strains (Supplementary Table 10, Supplementary Fig. 3). One of these genes is similar to the transposase in the DNA transposon *Sly1-1*^39^ and a second one is found next to this gene in both *Sk-2* and *Sk-3*, suggesting that they may form a single TE together. The third gene shows no homology to any genes with known function. Our data also suggest that the rate of gene gains in the spore killer region is not higher than in the homologous region of the sensitive strains of *N. intermedia* and *N. crassa*.

A region necessary for killing was recently identified in an *Sk-2* killer strain^28^. This region contains an 11 kb insertion that is located between the inverted region and the collinear region at the right edge of the *Sk-2* region (Fig. 1) and that is only found in the four *Sk-2* strains. A smaller part of this insertion has recently been found to be the active killer factor^29^, and when using nucleotide *blast* to find homologous sequences in the *Sk-3* genome, we were unable to find a similar sequence, neither at the homologous location nor anywhere else in the genome. However, by searching for the translated amino acid sequence, we identified a sequence on the left arm of chromosome 3 in both the *Sk-3* strain and several sensitive *N. intermedia* strains that gave a weak hit (*E*-value 0.007). We performed a knockout of this sequence in a *N. crassa* strain carrying the *Sk-3* region^26,30^, but no loss of killing was observed when crossing the knockout strain to a sensitive *N. crassa* strain (Supplementary Figs. 4 and 5). This result suggests that different genes are responsible for killing in *Sk-3* and *Sk-2* strains.

### The population structure of *N. intermedia*

We sequenced 21 *N. intermedia* strains, including all known *Sk-2* and *Sk-3* strains and 5 *N. metzenbergii* strains (Supplementary Table 11) using the Illumina HiSeq platform. We then called SNPs, which were used to investigate the population structure of *N. intermedia* and to compare the evolutionary history of *Sk* region to that of the background genome (i.e., all chromosomes except chromosome 3). Previous studies of *N. intermedia*, based on four molecular markers, have suggested that the species is dominated by two major phylogenetic subgroups that are clearly diverged from each other and show low levels of diversity^40^. Among the 21 strains analyzed here, only 6 cluster with one of these 2 subgroups (Supplementary Fig. 6a), the remaining strains show long branches and weak clustering, indicating that the earlier studies failed to capture the diversity within *N. intermedia*. Three of the five *N. metzenbergii* strains were originally annotated as *N. intermedia*, but based on the clustering here we reclassify them as *N. metzenbergii*.

Except in the case of strain 7426, the original isolates of the spore killer strains are no longer available and 4 out of the 5 known spore killer strains instead remain as 1–3 times backcrosses to an *N. intermedia* tester strains from Taiwan (in this study represented by strain 8761)^26^ (Supplementary Table 1). This prevents a phylogenetic analysis of the natural genetic background of the spore killers, and only the signal of the backcrossing is apparent in the clustering (Supplementary Fig. 6a).

### An old evolutionary separation of *Sk-2* and *Sk-3*

**Fig. 4:**
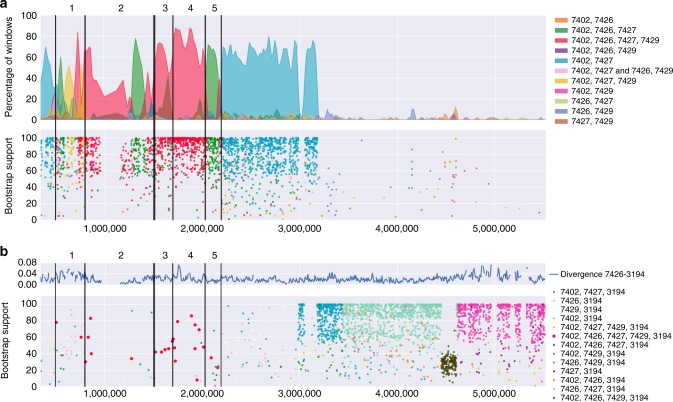
Phylogenetic signal over chromosome 3. SNPs from all 21 *N. intermedia* and 5 *N. metzenbergii* strains were split into non-overlapping windows each containing 50 variable sites, and a phylogenetic tree was inferred using the BioNJ algorithm in PhyML for each window. **a** The windows were then classified based on which of the four *Sk-2* strains were grouping together in the tree. All *Sk-2* strains share inversions 1–5 and in the absence of recombination the entire non-recombining region should form a monophyletic cluster (shown in red). When three or fewer *Sk-2* strains group together, this indicates that recombination has happened (other colors). Top panel show the percentage of each monophyletic grouping in bins of 50 trees. The bottom panel shows the bootstrap support for the monophyletic group identified in each tree. Black bars show the borders of the five major inversions. In inversions 3 and 4 (as indicated by the numbers above the plot), the main signal is of all four strains grouping together, indicating no recombination. In inversion 5 and in a section of inversion 2, strain 7429 no longer groups with the other three (marked in green), consistent with a recombination event. The breakpoints of all inversion show the red grouping, indicating that the recombination events did not span several inversions. **b** Phylogenetic signal of monophyly of *Sk-3* together with *Sk-2*. Top panel shows pairwise divergence between *Sk-2* strain 7426 and *Sk-3* strain 3194, in 10 kb sliding windows with a 2 kb step. Bottom panel show windows where *Sk-2* and *Sk-3* form a monophyletic group. Colors indicate which *Sk-2* strains (7492, 7426, 7427, 7429) group with *Sk-3* (3194) and the *y* axis indicates bootstrap support for that specific group. In the non-recombining region of chromosome 3, no strong signal for monophyly could be detected with only a smaller number of low bootstrap windows. The large clusters of windows on the right which show a signal of *Sk-2*-*Sk-3* monophyly are the product of the backcrossing of the spore killer strains to the Taiwan genetic background

The inversions present in the *Sk* region are expected to suppress recombination and potentially prevent gene flow between sensitive and killer strains, and accordingly, earlier studies have reported extremely low recombination rates within the spore killer regions^26,27^. We used a phylogenetic sliding window approach to investigate signatures of recombination between *Sk-2* strains and sensitive strains occurring after the establishment of the inversions, searching for regions where the four *Sk-2* strains do not show a monophyletic relationship. This analysis revealed several putative within-inversion, double-crossover events within the spore killer region (Fig. 4a). While inversions 3 and 4 (Supplementary Table 2) harbored no recombination events, *Sk-2* strain 7429 shows signs of recombination throughout most of inversion 5 and in a large region of inversion 2. In inversion 1, recombination appears to be more common, with a noticeably weaker signal of monophyly as compared to the other inverted regions. Only inversions 3 and 4 and parts of inversions 1 and 2 show a signal of monophyly for all four *Sk-2* strains, consistent with a shared phylogenetic history since the establishment of the suppression of recombination.

Recombination between the four *Sk-2* strains was also evaluated. No larger crossover tracts could be identified but a couple of smaller regions, potentially corresponding to gene conversion events, showed signal of genetic exchange (Supplementary Fig. 7).

Using the same phylogenetic sliding window approach, we find no strong signal of monophyly between the *Sk-2* and *Sk-3* strains in the inverted region (Fig. 4b). Furthermore, *Sk-3* groups on its own in a phylogenetic analysis of both the whole inverted region and the part where all *Sk-2* strains are monophyletic (Supplementary Fig. 6b and 6c), indicating that *Sk-3* and *Sk-2* are of independent origin.

Finally, we estimated the age of *Sk-2* based on the synonymous substitution rate. Pairwise synonymous divergence was calculated per gene between the *Sk* strains and all sensitive *N. intermedia* strains in the monophyletic regions. Since the sensitive strains are expected to be recombining freely, we could not identify a single sensitive strain that is the closest relative to the *Sk* strains, and instead we selected the gene copy that showed the least amount of divergence for each gene. This strategy allowed us to calculate a lower bound for the age of the non-recombining region to be between 255,000 and 428,000 years (Supplementary Table 12).

### Reduced efficacy of selection in the *Sk-2* region

In order to investigate the evolutionary trajectory of the *Sk* regions, we compared the diversity and divergence of non-synonymous and synonymous substitutions within the *Sk* region and in the rest of the genome. This analysis could only be performed for the sensitive and *Sk-2* strains, since we only have a single *Sk-3* strain in our dataset.

For the sensitive strains of *N. intermedia*, the nucleotide diversity varied little between chromosomes (*π*_total_, *π*_N_, and π_S_). The *π*_N_/*π*_S_ ratio also showed marginal changes across chromosomes with values ranging from 0.18 to 0.21 (Supplementary Table 13). The estimates for the *Sk* region of the sensitive strain was not significantly different from the rest of the genome, and no significant difference in sequence divergence to the outgroup, *N. metzenbergii*, was found. The Neutrality Index (NI) quantifies the direction and degree of departure from neutrality and was calculated as the ratio of *P*_N_/*P*_S_ over *D*_N_/*D*_S_. NI > 1 indicates negative selection while a value <1 indicates positive selection. In the sensitive strain, NI was close to 1 for the *Sk* region (1.04) as well as for the rest of genome (1.09), which suggests that the assumption of neutrality holds genome-wide. The summary statistics for nine sensitive strains gave similar results (Supplementary Table 14).

In contrast, the *Sk* region of the *Sk-2* strains differed significantly from the rest of the genome, with respect to both diversity and divergence from *N. metzenbergii* (Table 3). Specifically, while all chromosomes of the *Sk-2* strains showed similar diversity levels to the sensitive strains, the *Sk*-2 haplotype had only 40% of the average diversity at non-synonymous sites and even less at synonymous sites (*π*_N_ = 0.0034, *π*_S_ = 0.013, *p* value « 0.001). Because the reduction in the *Sk* region was more pronounced for synonymous sites, the *π*_N_/*π*_s_ ratio rose to 0.27, a value significantly higher than in the rest of the genome (0.19) (*p* value « 0.001). The *Sk-2* haplotype also had significantly more fixed divergent alleles than the rest of the genome (*D*_N_ = 2985 vs 2216, *D*_S_ = 7360 vs 4918, *p* values « 0.001). The rise of divergence in the *Sk-2* haplotype had a slightly stronger effect on synonymous sites than on non-synonymous sites, which led to a slight decrease of *D*_N_/*D*_S_ (*p* value = 0.14). The NI was higher but the difference was small and not significant (1.27, *p* value = 0.22). All measures of significance mentioned here were derived from comparisons of estimates from the *Sk* region to a null distribution bootstrapped from the rest of the genome (see Methods for further details).

**Table 3:** Nucleotide diversity and divergence of two *N. intermedia Sk-2* strains

*N. intermedia Sk-2* ^a^	*π*_total_	*π*_N_	*π*_S_	*π*_N_/*π*_S_	*P*_N_	*P*_S_	*D*_N_	*D*_S_	*P*_N_/*P*_S_	*D*_N_/*D*_S_	NI
Total	0.013	0.0077	0.041	0.189	58,830	153,013	59,077	132,834	0.38	0.44	0.86
Chr1	0.011	0.0062	0.033	0.185	12,349	31,767	14,403	30,655	0.39	0.47	0.83
Chr2	0.012	0.0079	0.042	0.188	6571	17,813	6003	13,624	0.37	0.44	0.84
Chr3^b^	0.017	0.0082	0.039	0.212	5293	13,168	4897	10,775	0.40	0.45	0.88
Chr4	0.013	0.0085	0.048	0.178	9922	26,513	8860	20,286	0.37	0.44	0.86
Chr5	0.019	0.0072	0.041	0.176	9006	25,031	8066	19,612	0.36	0.41	0.87
Chr6	0.013	0.0098	0.050	0.196	7171	18,260	6472	14,955	0.39	0.43	0.91
Chr7	0.012	0.0096	0.047	0.203	7748	18,962	7391	15,567	0.41	0.47	0.86
*Sk* reg.	0.003	**0.0034**	**0.013**	**0.265**	**770**	**1499**	**2985**	**7360**	**0.51**	0.41	1.27
Bootstrap mean (95%)^c^	⧸	0.008 (0.0067, 0.0092)	0.041 (0.038, 0.045)	0.19 (0.164, 0.2158)	2286 (1951, 2667)	5959 (5497, 6553)	2216 (1914, 2533)	4918 (4564, 5356)	0.384 (0.335, 0.432)	0.45 (0.4, 0.51)	1.17 (1.05, 1.31)

^a^Haplotypes were selected for calculation of diversity and divergence

^b^Chromosome 3 with spore killer region excluded

^c^Bootstrap confidence intervals were calculated by randomly picking the same number of genes as the number of genes in the Spore killer region. For bootstrap in total number of polymorphism and divergence, we also rescaled the number by the ratio of total bootstrapped gene length and the length of *sk* region. Significant values of *Sk* region are highlighted in bold

In summary, the sensitive strains evolved neutrally and the region homologous to the non-recombining region in the *Sk-2* strains did not depart in this respect from the rest of the genome. The increase in *π*_N_/*π*_s_ ratio also suggests that purifying selection was less effective in the *Sk-2* haplotype due to reduced recombination^41^.

An analysis of all variable sites was also performed (Supplementary Table 13, Supplementary Fig. 8). This supports the above analysis and shows that diversity is reduced in the *Sk* region. Linkage disequilibrium is elevated in the region but not consistently so, in agreement with occasional recombination between *Sk-2* strains.

### The *rsk* gene phylogeny

**Fig. 5:**
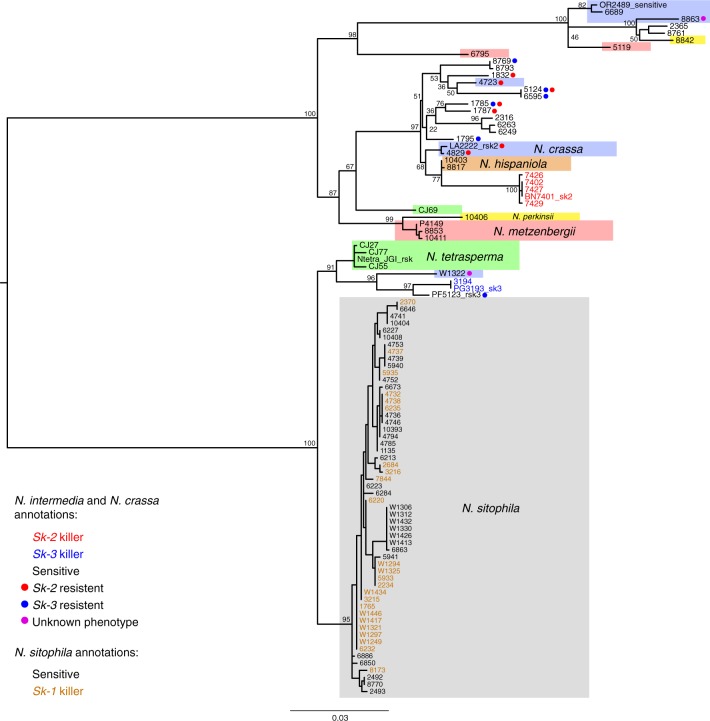
Maximum likelihood phylogeny of the resistance locus *rsk*. The alleles are split into two major groups, with *Sk-2* (marked with red text) in one and *Sk-3* (blue text) in the other. *Sk-2*-resistant strains (marked with red dots) cluster with *Sk-2*, but *Sk-3*-resistant strains (blue dots) are found in both groups. All *Neurospora sitophila* strains have similar alleles at the *rsk* locus, and there is no clustering depending on whether they are *Sk-1* killers (orange text) or sensitives (black text). Most *N. intermedia* and *N. crassa* strains have been tested for resistance against *Sk-2* and *Sk-3*, but this is not possible in most other *Neurospora* species, due to poor sexual compatibility. An exception is *N. metzenbergii*, where spore killing is observed in crosses with included strains

We performed a phylogenetic analysis of nearly 100 alleles of the *rsk* gene, previously shown to confer resistance against both *Sk-2-* and *Sk-3*-based killing^29^. *rsk* alleles from all *N. intermedia* strains included in this study and from several other *Neurospora* species were analyzed. The alleles form two major clusters (Fig. 5), which do not reflect the expected species relationship^31^. Strains of *N. intermedia*, *N. crassa*, and *N. tetrasperma* are found in both clusters, which indicates either ancestral polymorphism of *rsk* or extensive introgression. The most striking observation from our analysis is that strains that are resistant to *Sk-3* but not *Sk-2* have an *rsk* allele that is more similar to *Sk-2*-resistant strains. This observation contradicts the previous model^30^ predicting the existence of three major clades of *rsk* alleles (*Sk-2*-type alleles with *Sk-2* resistance, *Sk-3*-type alleles with *Sk-3* resistance, and *Sk-*sensitive alleles). An alignment of representative *rsk* alleles also shows that there are no polymorphisms in the gene that are unique for each resistance phenotype (Supplementary Fig. 9). Our findings suggest that *rsk*-based resistance to spore killing is more complicated than previously thought and that other genes may also influence the specificity of the resistance to each killer.

## Discussion

Meiotic drive is often associated with genomic divergence, both on large and small scales^1^, but the size and complexity of MDEs has hitherto impeded detailed analyses of the genomic regions harboring them. Here we generated high-quality genomic and epigenomic data of two different MDEs in the fungus *N. intermedia*. This dataset allowed us to investigate a range of changes associated with meiotic drive, from large-scale rearrangements to gene content, repeat distribution, heterochromatin patterns, and signals of molecular divergence and degeneration. The genomic characteristics of the *Sk-2* and *Sk-3* haplotypes are similar to those observed in the *SD complex*^7^ and the *t haplotype*^42^, with both an accumulation of inversions and signs of degeneration, but our data have allowed us to study these changes in greater detail, and they have also revealed that similar changes have taken place independently in the two different spore killer systems.

*Sk-2* and *Sk-3* will both produce four-spored asci when crossed to a sensitive strain, and it is first when they are crossed to each other and mutual killing is observed that it becomes clear that they are functionally distinct. Despite being located in the same part of the genome, they are also genetically distinct and appear to have originated independently or at least have experienced a long evolutionary separation. This conclusion is drawn based on three main observations: (1) *Sk-2* and *Sk-3* do not share any inversions, (2) a phylogenetic analysis of the non-recombining region do not show a signal of common descent for the two spore killers, and (3) the sequence responsible for killing in *Sk-2* cannot be found in the *Sk-3* genome. If *Sk-2* and *Sk-3* had a shared evolutionary history, we would expect them to share at least some of the inversions and give a stronger signal of relatedness. Instead, our observations suggest convergent evolution of two distinct MDEs that have evolved independently in the same genomic region.

A clue to why both *Sk-2* and *Sk-3* are located in the same region may be that they share the resistance gene, at which they carry different alleles that confer specific resistance to their respective killing mechanisms. The resistance gene is of unknown function, but specific alleles are thought to be able to neutralize the toxic activity of the spore killer genes^30^. The different alleles are highly diverged and show signs of ancestral polymorphism, which may have allowed distinct mutations causing spore killing to independently pair up with already existent resistance alleles that only confer resistance specifically to them. These mutations could have happened in different genes in different parts of chromosome 3, after which modifiers that suppress recombination between the killer and the resistance genes, such as inversions^43^, have evolved, generating the genome structure that we see in our data. An alternative hypothesis for the evolution of *Sk-2* and *Sk-3* is that they have evolved from a common, ancestral, spore killer haplotype, where recombination suppression was achieved through non-structural means, which would relax selection against inversions. Such large-scale suppression of recombination without structural rearrangements was recently shown for the mating-type chromosomes in *N. tetrasperma*, and it was concluded that the large inversions found in these regions are derived and not the cause of the suppression^32^. We cannot with our data formally exclude this hypothesis, but the weak signal of monophyly of *Sk-2* and *Sk-3* and the fact that no homologous sequence to the *Sk-2 rfk-1* killer sequence can be found in *Sk-3* indicates that this is a less likely scenario.

A complete understanding of the molecular basis for killing in *Sk-2* and *Sk-3* is not yet available. A sequence required for killing, called *rfk-1*, was mapped to the right flank of *Sk-2*^28^, corresponding to positions ~2,700,000–2,745,000 in the *Sk-2* 7426 haplotype. This region has been carefully dissected with gene deletion and complementation experiments, and the existence of a genetic element required for killing has been confirmed^29^. Utilizing our comparative transcriptomic approach, we were able to annotate the killer haplotypes and by doing so reveal a small number of novel ORFs in the region. Future functional analyses will reveal whether any of these genes are critical components of the killing and resistance mechanism or auxiliary modifiers of the meiotic drive system.

The inversions identified in the *Sk-2* and *Sk-3* haplotypes may be the cause of the suppression of recombination, and the fact that the inversions in the *Sk-2* strains cover the exact region from the resistance gene to the killer sequence is suggestive of this. Indeed, the fact that *Sk-2* and *Sk-3* both carry inversions suggests that these are beneficial in linking together the killer and resistance genes. It is at this point not possible to determine whether the recombination suppression is caused by the inversions or by non-structural means as in *N. tetrasperma*^32^, but since any odd number of crossovers between the killer and the resistance loci will be highly deleterious, selection for reducing recombination is likely to be strong and both structural and non-structural mechanisms may act additively to reduce the recombination rate as much as possible.

It is possible that the inversions were facilitated by repetitive sequences, which also have accumulated in the *Sk* haplotypes. We see a strong increase of TEs in the *Sk* regions and this can be explained by several processes. Genome defence mechanisms such as RNA interference, heterochromatin, and RIP are known to limit the spread of TEs^44^, as is the ability to purge TEs through recombination with non-carrier strains^45^ or through recombination-associated unequal crossovers^46^. If these defence mechanisms are impaired or if recombination is reduced, the total TE content of a genome may increase, as is the case if the genome is invaded by new TE families that can evade the genome defence. We took advantage of the knowledge of the broad and efficient genome defence systems and repeat content in *Neurospora*^34,47^ to evaluate the relative importance of the different processes and detected no signs of either weakened genome defence mechanisms or an influx of new TE types. Specifically, a comparable GC distribution in repetitive and non-repetitive regions between spore killer strains and other *Neurospora* strains indicates a conserved RIP machinery^35^, while ChIP- and bisulfite sequencing data suggest that patterns of heterochromatin are normal. Instead the suppression of recombination of the region may allow repeats to accumulate due to a weaker efficiency of selection, i.e., slightly deleterious repeats cannot be purged because they are completely linked to the killing and resistance genes^48^. This hypothesis is consistent with the molecular evolution data and reduced *D*_N_/*D*_S_ values of the *Sk-2* haplotype, for which population-level analyses were possible. Because this region shows suppression of recombination with sensitive strains and because killer strains (with which they can freely recombine) appear to be rare, our results on reduced diversity and elevated *π*_N_/*π*_S_ ratio of the spore killer region are in line with expectations of a reduced efficiency of selection in this region^41^. These findings are consistent with the recessive lethal mutations identified in the *t haplotype*^42^, except that the mutations accumulated in *Sk-2* and *Sk-3* would directly affect viability, due to the fact that *N. intermedia* grows vegetatively in the haploid state. Fitness assays of isogenic strains with and without the *Sk* haplotypes are needed in order to determine whether there is a more subtle negative fitness effect of these mutations.

Similar patterns of molecular evolution as found herein are found in other supergene systems, where coadapted gene complexes inhabit regions of suppressed recombination. For instance, molecular degeneration and large expansions of repetitive sequences are seen in animal and plant sex chromosomes^49^ and in many fungal mating-type chromosomes^14,45^. In many of these cases, degeneration has gone much further, with extensive loss of both genes and synteny^12,50^, but the conserved synteny and limited signs of gene loss found for *Sk-2* is consistent with it being comparatively young (250–430 thousand years). We also see parallels between our MDEs and supergenes in convergent evolution for rearrangements and suppression of recombination^11,51^, supporting the idea that the need to link co-evolving genes may be an important driver of genome structure. On the other hand, both *Sk-2* and *Sk-3* have only very rarely been found in nature, whereas the single-gene spore killers in *P. anserina* and *S. pombe* appear to be much more common^23–25^. This distribution pattern suggests that either multilocus systems are more complex to evolve or that they fail to reach high frequencies quickly enough as the degeneration associated with suppressing recombination prevent them from spreading in the population.

Our dataset has a number of limitations. First, having only a single *Sk-3* strain available means that we cannot detect signals of molecular degeneration in the non-recombining region of this spore killer, and we cannot test if it has experienced recent recombination. Furthermore, the backcrossing of four out of five spore killers to a laboratory strain means that we have a reduced ability to study the background genome of these strains and determine whether it differs from that of sensitive strains. For instance, *Sk-2* could potentially form its own subpopulation of *N. intermedia* that only rarely interacts with sensitive strains. However, the fact that we detected evidence of recombination between *Sk-2* and sensitive strains but limited signs of recombination among *Sk-2* strains argues against this hypothesis.

In summary, this study is the first example of a complete assembly of a complex MDE that spans a large section of a chromosome, allowing the evaluation of the association between meiotic drive and genome architecture at an unprecedented resolution. Our data show a strong local impact of meiotic drive on chromosome architecture. It also suggests that TEs may be working in concert with meiotic drive in that they facilitate inversions and that suppression of recombination allows them to accumulate in the region, resulting in the killer haplotype having a different evolutionary trajectory than the rest of the *Neurospora* genomes.

## Methods

### Strains included in the study

All strains used in this study were ordered from the Fungal Genetics Stock Center (http://www.fgsc.net)^52^ and are listed in Supplementary Table 1. All strain used in this paper are referred to by their FGSC identification number. The reported phenotypes (i.e., *Sk-2*, *Sk-3*, sensitive or resistant) were confirmed by crossing them to *N. intermedia* spore killer tester strains. Crosses were performed on SC medium^53^ and each strain was crossed to an *Sk-2* (7427 *mat A*, 7428 *mat a*), an *Sk-3* (3193 *mat A*, 3194 *mat a*), and a sensitive (1766 *mat A*, 1767 *mat a*) strain of the opposite mating type. Only strains that showed the proper phenotype in all three crosses were included in the study.

### Generation of genomic data using PacBio sequencing

Whole genomes of six strains (Table 1) were sequenced using the PacBio RSII platform (Pacific Biosciences). The strains were cultured by inoculating 500 ml Erlenmeyer flasks containing 200 ml of liquid malt extract (3%) medium with conidia (asexual spores). The flasks were incubated at 30 °C on a rotary shaking table for 3–4 days, and the cultures were then harvested by removing the mycelium from the flask and squeezing it between filter papers to remove excess liquid. The harvested mycelium was cut into small pieces and approximately 1 g was allotted into 2 ml tubes with screw-on caps, after which the tubes were stored at −20 °C until extraction. To extract the DNA, tissue of two tubes of each strain were freeze-dried overnight and macerated using a TissueLyzer II bead-beater (Qiagen). Two 2 mm metal beads were placed in each tube, which were then shaken at 25 Hz for 20–40 s, until no larger fragments remained. DNA was extracted using Genomic Tip G-500 columns (Qiagen) and cleaned using the PowerClean DNA Clean-Up Kit (MoBio Labs). The extracted DNA was sent to the Uppsala Genome Center (Science for Life Laboratory, Uppsala, Sweden), where libraries were prepared and sequenced on a PacBio RSII system, using four SMRT cells per sample and the C4 chemistry and P6 polymerase (Pacific Biosciences). Raw PacBio sequence data was filtered and assembled using the SMRT Analysis package and HGAP 3.0 assembler (Pacific Biosciences, https://github.com/PacificBiosciences/).

### Generation of genomic data using Illumina HiSeq sequencing

Whole genomes of 91 strains were sequenced with Illumina HiSeq technology (Supplementary Table 1). For these samples, conidia were inoculated into 50 ml plastic culture tubes containing 10 ml of liquid malt extract (3%) medium. The tubes were incubated at 30 °C on a rotary shaker for 2–3 days before harvesting, which was done by removing the mycelium from the culture tubes and squeezing it between filter paper to remove excess liquid. The mycelium was then cut into small pieces and approximately 100 µg of tissue was allotted into 1.5 ml Eppendorf tubes, which were stored at −20 °C until extraction. Whole-genome DNA was extracted using the Fungal/Bacterial Microprep Kit (Zymo, www.zymo.com) and sent to the SNP&SEQ Technology Unit (SciLifeLab, Uppsala, Sweden), where libraries were prepared and sequenced in four lanes on an Illumina HiSeq 2500 system. Illumina reads were assembled de novo using ABySS^54^, with the following parameters: kmer size = 64, bubble size = 3, and minimum contig size = 200.

### Fungal tissue for RNA-, bisulfite-, and ChIP-sequencing

We gathered transcriptome, bisulfite, and ChIP-seq data from three strains of *N. intermedia*, an *Sk-2* strain, an *Sk-3* strain, and a sensitive strain (Supplementary Table 1). For tissue collection during the vegetative development, strains were grown on solid Vogels medium N (Vogel 1956), on 90 mm Petri dishes covered with cellophane. After 2 days of growth at 25 °C, under 12:12 light–dark conditions, hyphal tissue was harvested from the surface of the cellophane with a sterile scalpel. Tissue from each plate was divided into three parts: One part of the tissue was stored at −20 °C for DNA extraction, a second part was immediately frozen in liquid nitrogen and stored at −80 °C for RNA extraction, and the third part of the tissue was immediately crosslinked with formaldehyde for the ChIP-seq experiment and stored at −80 °C. Three biological replicates were included by growing each strain on three plates.

In addition, transcriptome data were gathered from crosses. Crosses were performed on a single layer of Miracloth (50 × 50 mm^2^, MilliporeSigma, 475855-1 R) over 30.0 ml of synthetic crossing medium (pH 6.5, 1.5% sucrose; Westergaard and Mitchell 1947) in 100 mm diameter petri dishes. FGSC 1767 (sensitive *a*) was inoculated to the center of each sheet of Miracloth and cultured for 6 days before fertilization with FGSC 1766 (sensitive *A*), FGSC 7426 (*Sk-2 A*), or FGSC 3193 (*Sk-3 A*). Fertilizations were performed by transferring approximately 500 µl of a freshly prepared conidial suspension to each culture of FGSC 1767 (*Sk*^*S*^ *a*). Crosses were incubated at room temperature (RT) on laboratory shelves under ambient light conditions until 36 h after the first ascospores appeared on the undersides of the lids of the petri dishes. At this time point, which occurred at 11 days post fertilization (dpf) for FGSC 1767 × FGSC 1766, 12 dpf for FGSC 1767 × FGSC 7426, and 13 dpf for FGSC 1767 × FGSC 3193, perithecia and surrounding vegetative tissue were harvested from the sheets of Miracloth with razor blades, squished between filter paper to remove excess moisture, and ground to a fine powder under liquid nitrogen with a mortar and pestle. Total RNA was first isolated from ground tissue with TRIzol Reagent (Invitrogen) following the manufacturer’s guidelines. Total RNA was then purified with the miRNeasy Kit (Qiagen) following the manufacturer’s protocol for total RNA isolation with an on-column DNAase digestion. For each cross, we performed three biological replicates.

### Bisulfite sequencing

For whole-genome bisulfite DNA sequencing, DNA was extracted using the ZR Fungal/Bacterial DNA MiniPrep™ Kit according to the manufacturer’s recommendations. Sequencing libraries were prepared from 100 ng of DNA using the TruSeq Methylation Kit (Illumina Inc., EGMK91324) according to the manufacturer’s protocol (#15066014). Unmethylated lambda DNA (Promega, D152A) was spiked-in at 0.1% to the samples in the library preparation procedure and sequencing was performed on Illumina HiSeq 2500 platform (SciLifeLab Uppsala) to generate 125 bp paired-end reads. All steps in the procedure were performed separately for the biological replicates.

### ChIP and sequencing

For histone modification ChIP, 2-day-old mycelia tissue was crosslinked using 1% formaldehyde in 1× phosphate-buffered saline (PBS: 137 mM NaCl, 2.7 mM KCl, 10 mM Na_2_HPO_4_, and 1.8 mM KH_2_PO_4_) with gentle shaking at RT for 30 min. The reaction was quenched by adding glycine to final concentration of 125 mM and incubation at RT with gentle shaking for 5 min. The collected mycelia were washed with PBS, and for each biological replicate, 100 mg was homogenized by pestle in 600 µl ChIP lysis buffer (16.7 mM Tris-HCl - pH 8.0, 167 mM NaCl, 1.2 mM EDTA, 1.1% Triton X-100, 1× cOmplete EDTA-free protease inhibitor coctail (Roche)). The homogenized tissue was disrupted by sonication with submerged probe for 5 cycles, 20 s ON/40 s OFF, 5% intensity. ChIP lysis buffer was added to samples to 1000 μl and chromatin was then sheared by 60 cycles of sonication of 30 s ON/30 s OFF using high mode on a Bioruptor® (Diagenode) to reach median DNA fragment size of 0.5–1 kb. The sheared chromatin suspension was cleared by centrifugation (14,000 rpm, 5 min, 4 °C). In all, 15 μl of chromatin was set aside as input control and 150 μl of chromatin were mixed with 2 μl of antibody for each immunoprecipitation reaction. The following antibodies were used: IgG from rabbit serum (1 mg/ml, Sigma, cat. no. I5006), anti-Histone H3 (N-terminal) (Sigma, cat. no. H9289), anti-H3K4me2 (Active Motif, cat. no. 39141), anti-H3K9me3 (Active Motif, cat. no. 39161), and anti-H3K27me3 (Millipore, cat. no. 07-449). After overnight incubation with rotation at 4 °C, the immunoprecipitated complexes were collected using 15 μl of Dynabeads® Protein A (Thermo Fisher Scientific) per reaction after incubation for 1.5 h with rotation at 4 °C. Beads were washed once in 150 μl of following buffers at 4 °C: ChIP lysis buffer (without protease inhibitor) for 10 min, high-salt ChIP lysis buffer (as described above but supplied with 0.5 M NaCl) for 10 min, LiCl buffer (20 mM Tris-HCl—pH 8, 1 mM EDTA, 1% sodium deoxycholate, 1% NP-40 and 0.25 M LiCl) for 5 min, followed by 5 min in TE buffer (10 mM Tris-HCL, pH 8.0, 1 mM EDTA). Chromatin reverse crosslinking and DNA elution from input and immunoprecipitated samples were performed using iPure Kit (Diagenode, C03010015) following the manufacturer´s instructions. Three immunoprecipitation reactions from independent biological replicates were processed for next-generation sequencing library preparation. Each library was made from 5 ng of DNA using the MicroPlex Library Preparation Kit (Diagenode, C05010014) following the manufacturer’s instructions. Pools of libraries mixed in equimolar amounts were loaded onto 1% agarose electrophoresis gel for size selection. Fragments of 250–750 bp were excised from the gel and DNA was purified using the QIAquick Gel Extraction Kit (Qiagen). All steps were performed separately for the three biological replicates.

### RNA sequencing

RNA was extracted from both vegetative and crossing tissue, prepared as outlined above. Traces of DNA were removed by DNase I treatment (Fermentas). The RNA concentration and quality was determined spectrophotometrically using Nano-Drop (Thermo Scientific), and RNA quality was assessed after electrophoresis on an Agilent Bioanalyzer using the RNA 6000 Nano Kit (Agilent Technologies, Santa Clara, CA) according to the manufacturer’s instructions. Sequencing libraries for mRNA were constructed by following the standard protocols of Illumina TruSeq mRNA library preparation kits, respectively (Cat# RS-122-2101/2102, Illumina Inc). Sequencing was performed on Illumina HiSeq 2500 platform at SciLifeLab Uppsala, to generate 125 bp paired-end reads. All steps were performed separately for the biological replicates.

### Deletion of RFK-1 homolog in *Sk-3*

The genome of *Sk-3* strain 3194 was searched with tblastn (BLAST 2.7.1+) for homologs of a protein required for *Sk-2*-based spore killing (RFK-1, 39 amino acid model). The most significant match to *Sk-2* RFK-1 was found between positions 187,891 and 187,783 of chromosome 3. Two deletions vectors were constructed to delete this region (and flanking sequences) from RDGR170.3, an Sk-3-harboring strain of *N. crassa*^55^. Deletion vectors were constructed by Double-Joint PCR^56^. Specifically, they were designed to replace the following positions with a hygromycin selectable marker: 185,356–188,445 (Vector 207) and 187,617–188,445 (Vector 208). Deletion strains were obtained by electroporation of washed conidia as previously described^57^. Homokaryotic transformants were isolated by the microconidium method of Ebbole and Sachs (http://www.fgsc.net/fgn37/ebbole1.html) and confirmed by PCR.

### Whole-genome alignments

To study structural variation in the PacBio assemblies, the MUMmer whole-genome aligner was used^58^. All genomes were aligned to the *N. crassa* OR74 assembly and to each other with the following parameters: nucmer –c 200 –b 2000. MUMmer alignment files were then visualized using a custom Python script.

### Analysis of methylated DNA

Paired-end Illumina HiSeq reads were mapped to PacBio-based de novo assemblies of three *N. intermedia* strains: 3194 (*Sk-3*), 7426 (*Sk-2*), and 8761 (sensitive). The reads were trimmed using trimGalore (http://www.bioinformatics.babraham.ac.uk/projects/trim_galore/) (parameters: trim_galore–paired–trim1), and then mapped to the genomes using Bismark (http://www.bioinformatics.babraham.ac.uk/projects/bismark/) (parameters: bismark -X 700 –un). The reads where then deduplicated using the deduplicate bismark tool and finally methylated sites were extracted using bismark_methylation_extractor (parameters: bismark_methylation_extractor–paired-end–comprehensive–cytosine_report–CX–bedGraph–report–gzip–ignore 12–ignore_r2 12–ignore_3prime 3–ignore_3prime_r2). Whole-chromosome methylation patterns were visualized by merging the triplicate samples and calculating the percentage of methylated sites in 2 kb windows using a custom python script.

### Analysis of ChIP-seq data

ChIP-seq reads were cleaned from adapter contamination and trimmed using CutAdapt^59^ and Trimmomatic^60^. The reads were then mapped to the corresponding PacBio assemblies using BWA^61^, deduplicated with Picard (http://broadinstitute.github.io/picard/), and mapping coverage was finally extracted using bedtools (https://bedtools.readthedocs.io/). Chromatin density was calculated as fold increase of coverage in immunoprecipitated samples compared to input control samples.

### Analysis of repetitive DNA

Repetitive DNA and TEs were called using RepeatMasker (http://www.repeatmasker.org) and a library of *Neurospora*-specific repetitive elements^47^. Repeat enrichment in the *Sk* haplotypes was calculated using a one-sided binomial test, with Bonferroni corrected significance thresholds. Repeat clusters were identified using a sliding window approach. Percentage of repetitive sequence was determined in 2 kb non-overlapping windows, and windows consisting of more than 75% repetitive sequence were classified as repetitive. DNA methylation, H3K9me3 coverage, and GC content were then also calculated per window, and the average levels of these characteristics among windows classified as repetitive, non-repetitive, and unclassified (all windows) was plotted.

### Analysis of transcriptome data

The raw reads from Illumina HiSeq were cleaned from adapter contamination and trimmed using CutAdapt and Trimmomatic as above. The trimmed reads were mapped to the high-quality PacBio assemblies using STAR^62^ (parameters: STAR–alignIntronMax 20000–alignMatesGapMax 20000–alignSJoverhangMin 10–outSAMtype BAM Unsorted–outFilterIntronMotifs RemoveNoncanonical–twopassMode Basic). The mapped reads were then assembled into transcripts using cufflinks^63^ (parameters:–library-type fr-firststrand). The data from the crosses were mapped to hybrid assemblies, where the non-recombining region of 7426 (*Sk-2*) or 3194 (*Sk-3*) was added to the 8761 (sensitive) assembly. The use of a hybrid assembly makes it possible to separate gene expression during meiosis in the non-recombining region of the spore killer strains from the gene expression of the homologous region in the sensitive strain. The called transcripts were then used to annotate the PacBio assemblies using MAKER^64^, which generated gene models that could be used to compare gene content between the sequenced species.

### Comparisons of gene content

The gene content of strains 3194, 7426, and 8761 and *N. crassa* OR74 were compared by using OrthoMCL^65^ to infer orthological relationships. Protein sequences were extracted from the MAKER genome annotations of strains 3194, 7426, and 8761 and protein sequences of the *N. crassa* OR74 assembly was downloaded from FungiDB^66^. These were compared and orthologous relationships were inferred by performing an all-to-all protein BLAST search and clustering the genes with OrthoMCL. Genes that were unique for each strain were extracted and the ones located in the *Sk* region were further blasted back to the genomic sequences of all four strains using genBlastG^67^, in order to verify that they had not been missed in the annotation process of the other genomes. Gene function for these was inferred using HMMer (https://www.ebi.ac.uk/Tools/hmmer/) and searching FungiDB (http://www.fungidb.org).

An ORF that is necessary for killing in *Sk-2* has been identified by the Hammond laboratory^29^: it is located at positions 2,744,002–2,744,118 on chromosome 3 in the genome assembly of strain 7426. We searched the genome of the *Sk-3* strain 3194 and the sensitive strains 8761 and 8807 for homologous sequences using NCBI blastn and tblastn.

### Calling of SNPs

Whole-genome raw Illumina HiSeq reads were cleaned from adapter contamination and trimmed using CutAdapt and Trimmomatic as above. The trimmed reads were then mapped to the PacBio assembly of the sensitive *N. intermedia* strain 8807^32^ using BWA. This strain was selected as the reference genome since all seven chromosomes were assembled into complete contigs. The BAM file produced by BWA was deduplicated with picard, and complex regions were realigned with GATK IndelRealigner^68^. SNPs were called using GATK (first using -T HaplotypeCaller -bamWriterType CALLED_HAPLOTYPES -stand_emit_conf 10.0 -stand_call_conf 20.0 -gt_mode DISCOVERY–emitRefConfidence BP_RESOLUTION, then merging all vcf files using -T GenotypeGVCF–sample_ploidy 1–includeNonVariantSites). Variants from regions that had been annotated as repetitive with RepeatMasker were removed together with sites with missing data, using VCFtools^69^. Finally, the VCF files were converted to fasta files using GATK VariantsToTable and a custom Python script. The final FASTA file contained all called sites.

### Phylogenetic analysis

The SNP data was used to infer phylogenetic relationships using RAxML^70^ with parameters: raxmlHPC-HYBRID-AVX -T 16 -m GTRCAT -x 45345 -p 22455 -# 100 -f a. Phylogenies for each chromosome were generated separately and were then merged together (excluding chromosome 3, which contains the *Sk* region) to form a network using Splitstree^71^. A phylogenetic network of the *Sk* region was also inferred by creating individual phylogenies for each inversion in the *Sk-2* strains individually and merging these with Splitstree.

The phylogeny of the resistance (*rsk*) gene was also inferred with RAxML using the same parameters. The *rsk* sequence was extracted from de novo assemblies of all strains included in this study, together with 50 *N. sitophila* assemblies, that have also been generated from Illumina HiSeq data and four *N. tetrasperma* genomes from ref. ^14^, using GenBlastG. The *rsk* gene sequence from the *N. tetrasperma*^72^ reference genome was also added to the dataset and four alleles sequenced by Hammond et al. (2012)^30^.

### Calculation of diversity and divergence in coding sequences

We calculated pairwise nucleotide difference on non-synonymous and synonymous sites (*π*_N_ and *π*_S_) for all protein-coding genes in both *N. intermedia* sensitive and *Sk-2* strains. Values were averaged across each chromosome except for chromosome 3, which was split into spore killer (*Sk*) and non-*Sk* regions. The *π*_N_/*π*_S_ ratio was then calculated as an indicator of purifying selection. We performed McDonald–Kreitman tests on the *Sk* region and on the rest of the genome. Owing to limited number of polymorphism and high linkage disequilibrium in the *Sk* region, we treated the *Sk* region as a whole chromosome and counted the total number of polymorphism and divergence at non-synonymous and synonymous sites (*P*_N_, *P*_S_, *D*_N_, *D*_S_). The neutrality index (NI = (*P*_N_/*P*_S_)/(*D*_N_/*D*_S_) was used as an indicator of positive/negative selection. For comparison, we performed the same analysis on each chromosome. To control for difference in gene number and sequence length, we carried out bootstraps on all statistics by randomly sampling the same number of genes as in the *Sk* region and rescaled by the sequence length. For all analyses, we chose two *Sk-2* strains (7427 and 7426) to reduce inbreeding effects introduced by several rounds of backcrossing to the Taiwan background^26^. To control for the variance introduced by sample size differences, we also carried out all analyses on a subsample of two randomly picked sensitive strains (1785 and 1787). We used two *N. metzenbergii* strains (6795 and P4149) as outgroup for calculation of divergence. All calculations were performed using the software dNdSpiNpiS_1.0 (http://kimura.univ-montp2.fr/PopPhyl).

### Analyzing recombination patterns

Recombination between *Sk-2* strains and other *N. intermedia* strains were assessed by creating phylogenetic trees from sliding windows of SNP data. The VCF files generated above were split up into sliding windows with a length of 50 variable sites, using the script phyml_sliding_windows.py downloaded from https://github.com/simonhmartin/genomics_general/ and phylogenies were generated using the BioNJ algorithm in PhyML (http://www.atgc-montpellier.fr/phyml/). Recombination was inferred by determining whether the four *Sk-2* were monophyletic or whether one or more *Sk-2* strain instead were grouping with other *N. intermedia* strains in the trees generated by PhyML. All trees were parsed and categorized using the Python library ETE Toolkit^73^ and the distribution over chromosome 3 was plotted.

Recombination between the four *Sk-2* strains was assessed using LDhat (https://github.com/auton1/LDhat), with the interval algorithm and theta = 0.001.

### Code availability

Scripts for analyzing recombination patterns and for calculating association between repetitive regions and heterochromatin are available at https://github.com/johannessonlab/intermedia_spore_killer/. Other scripts are available upon request.

## Electronic supplementary material

Supplementary information

## Data Availability

All raw sequencing reads generated in this study have been deposited at the Sequence Read Archive as BioProject PRJNA486257. Genome assemblies based on PacBio reads and of Illumina HiSeq read, SNP data in VCF format, predicted genes, and the alignment of the rsk gene have been deposited at Figshare (10.6084/m9.figshare.c.4202669)^74^. All other relevant data are available upon request.
