# Supplementary material for: Convergent evolution of complex genomic rearrangements in two fungal meiotic drive elements
Source: Nat Commun. 2018 Oct 12;9:4242. doi: 10.1038/s41467-018-06562-x (PMC6185902; doi:10.1038/s41467-018-06562-x)
Supplement: Supplementary file 1 — Supplementary information [file 41467_2018_6562_MOESM1_ESM.pdf]

# Supplementary Information

## Convergent evolution of complex genomic rearrangements in two fungal meiotic drive elements

Svedberg et al.

**Supplementary information includes:**

Supplementary figure 1-9
Supplementary table 1-14
Supplementary references

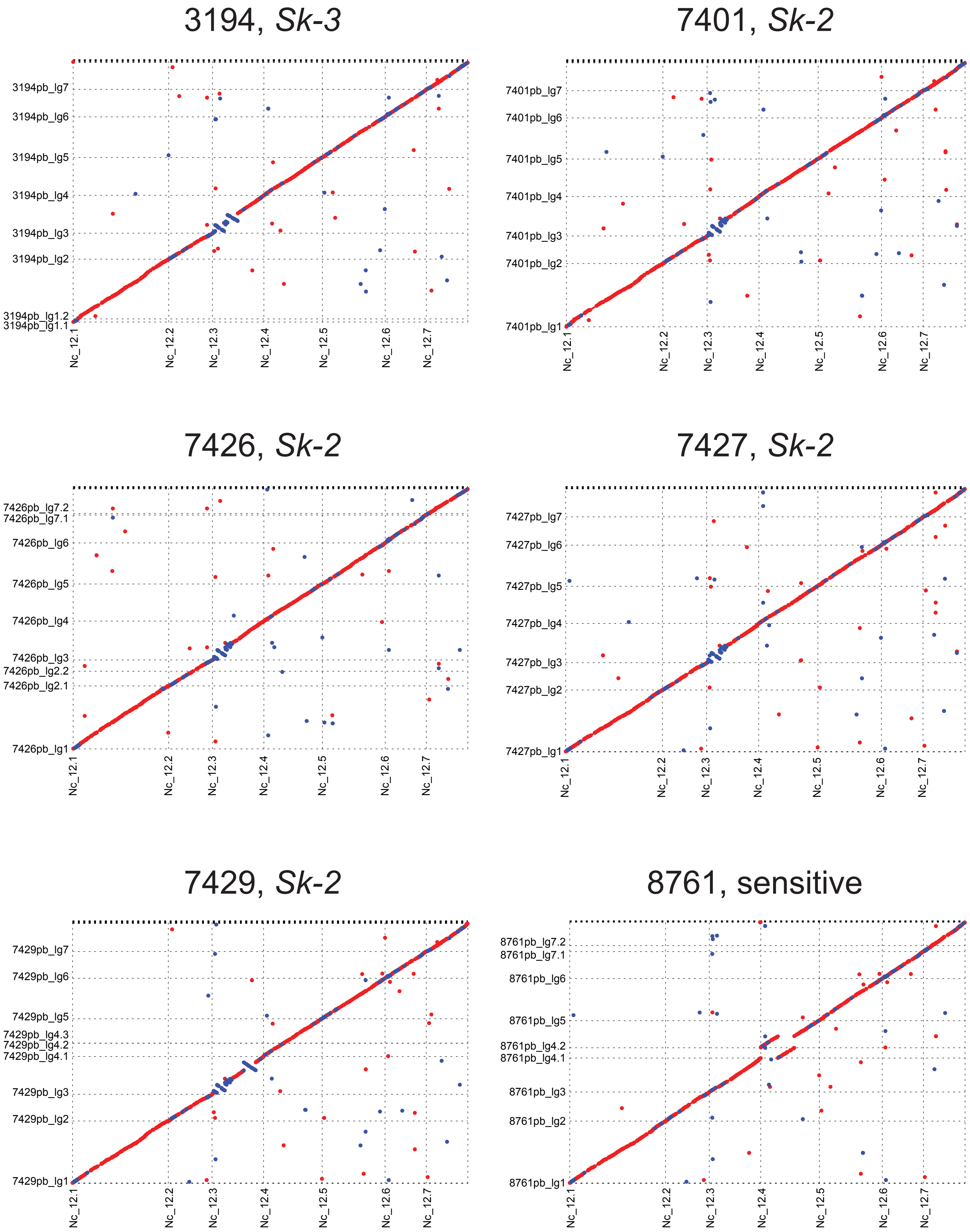


**Supplementary Figure 1**

Dotplots of the six PacBio assemblies aligned to the *N. crassa* OR74 genome. PacBio contigs are plotted vertically, and the *N. crassa* chromosomes horizontally.

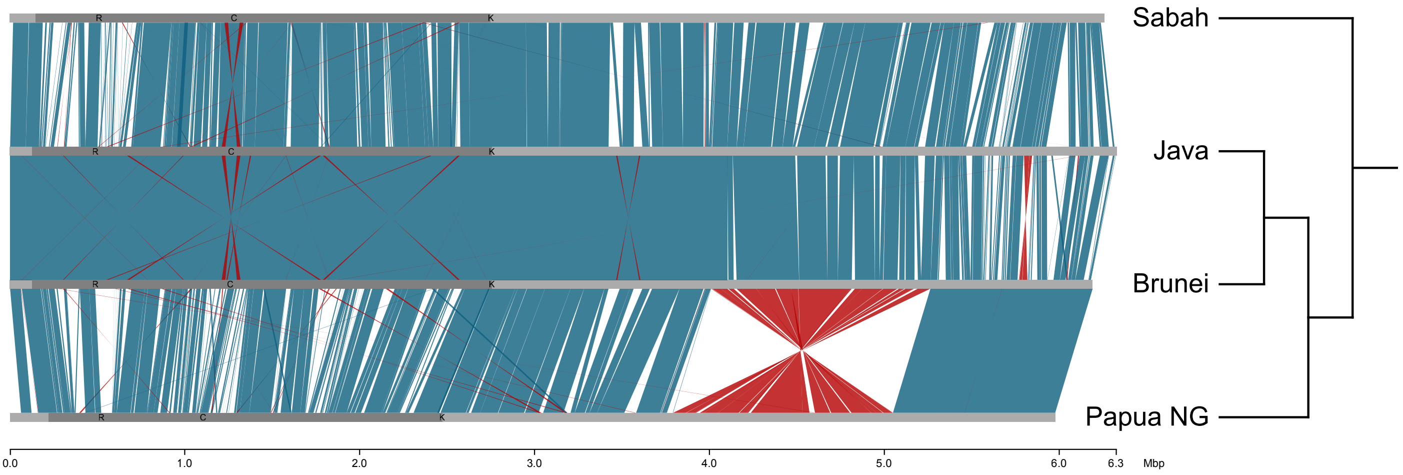


**Supplementary Figure 2**
Chromosome 3 of all four known *Sk-2* isolates were aligned to each other. Green blocks show collinear alignments and red blocks show inverted alignments. The dark grey bars indicate the region where the four *Sk-2* isolates all carries inversions relative to sensitive strains. The tree to the right shows their phylogenetic relationship. The inverted region of the Brunei and Java isolates are very closely related and show no structural differences. Comparing to the other isolates variation in repeat regions is clearly visible. The inverted region of the Papua New Guinea isolate is smaller and also carries an extra, large inversion to the right. Strains: Brunei = 7401, Sabah = 7426, Java = 7427, Papua New Guinea = 7429.

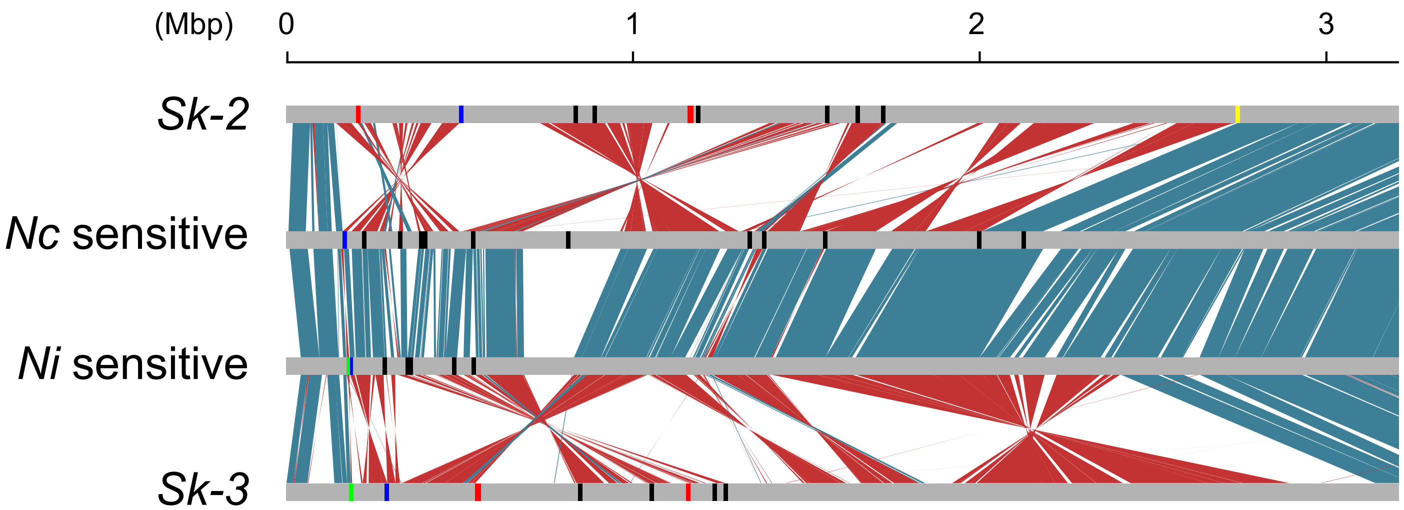


**Supplementary Figure 3**

Selected and unique genes in the *Sk* region. The genome assemblies of one *Sk-2* (7426), one *Sk-3* (3194) and one sensitive ("*Ni* sensitive", strain 8761) *N. intermedia* strain were annotated using transcriptomic data. The gene content of these assemblies were compared to each other and to the well-annotated sensitive *N. crassa* OR74 reference genome ("*Nc* sensitive") in order to identify genes in the *Sk* region that were unique for each strain (black vertical lines). Genes that were only found in the Sk-2 and Sk-3 genome assemblies and not in any of sensitive strains were also identified (red vertical lines). For reference, the *rsk* resistance locus is marked in blue and the *Sk-2 rfk-1* killer locus is marked in yellow. The sequence which showed homology to *rfk-1*, that was deleted in *Sk-3* is also found in the *N. intermedia* sensitive strain and is marked in green.

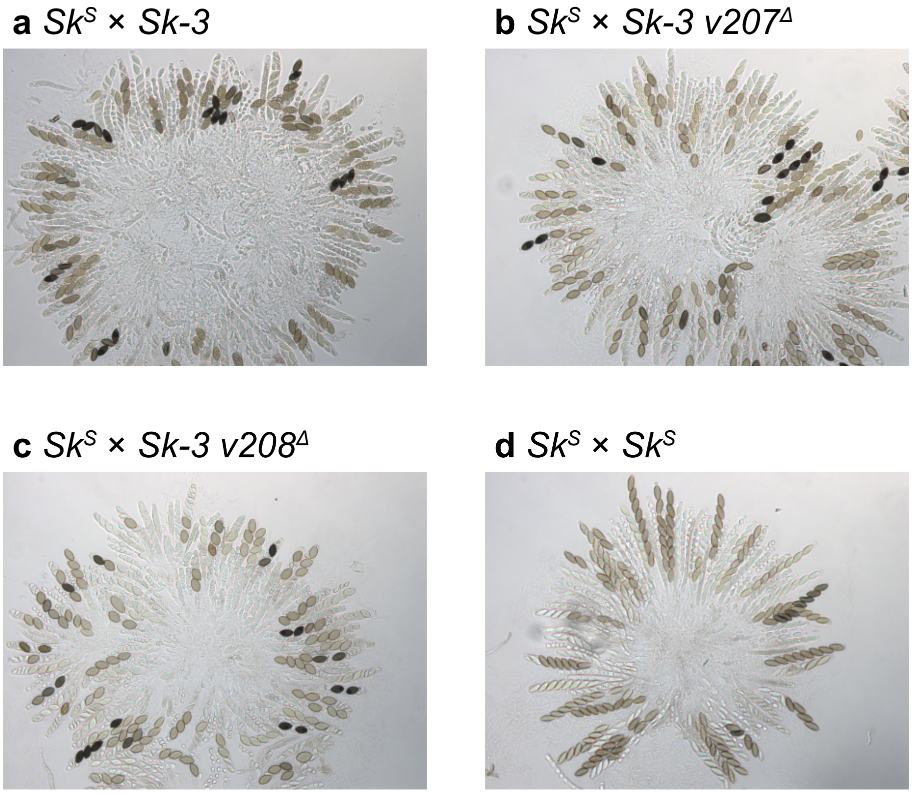


**Supplementary Figure 4**
Asci were dissected from perithecia 12 days post fertilization. Crosses are as follows: **a)** RTH1005.2 × RDGR170.3; **b)** RTH1005.2 × HAA207.6.1; **c)** RTH1005.2 × HAA208.4.1; **d)** RTH1005.2 × RTH1005.1.

*Genotypes*:
RTH1005.2: rid; fl; a
RTH1005.1: rid; fl; A
RDGR170.3: rid; Sk-3; mus-51Δ::bar A
HAA207.6.1: rid; Sk-3 v207Δ::hph; mus-51Δ::bar A
HAA208.4.1: rid; Sk-3 v208Δ::hph; mus-51Δ::bar A

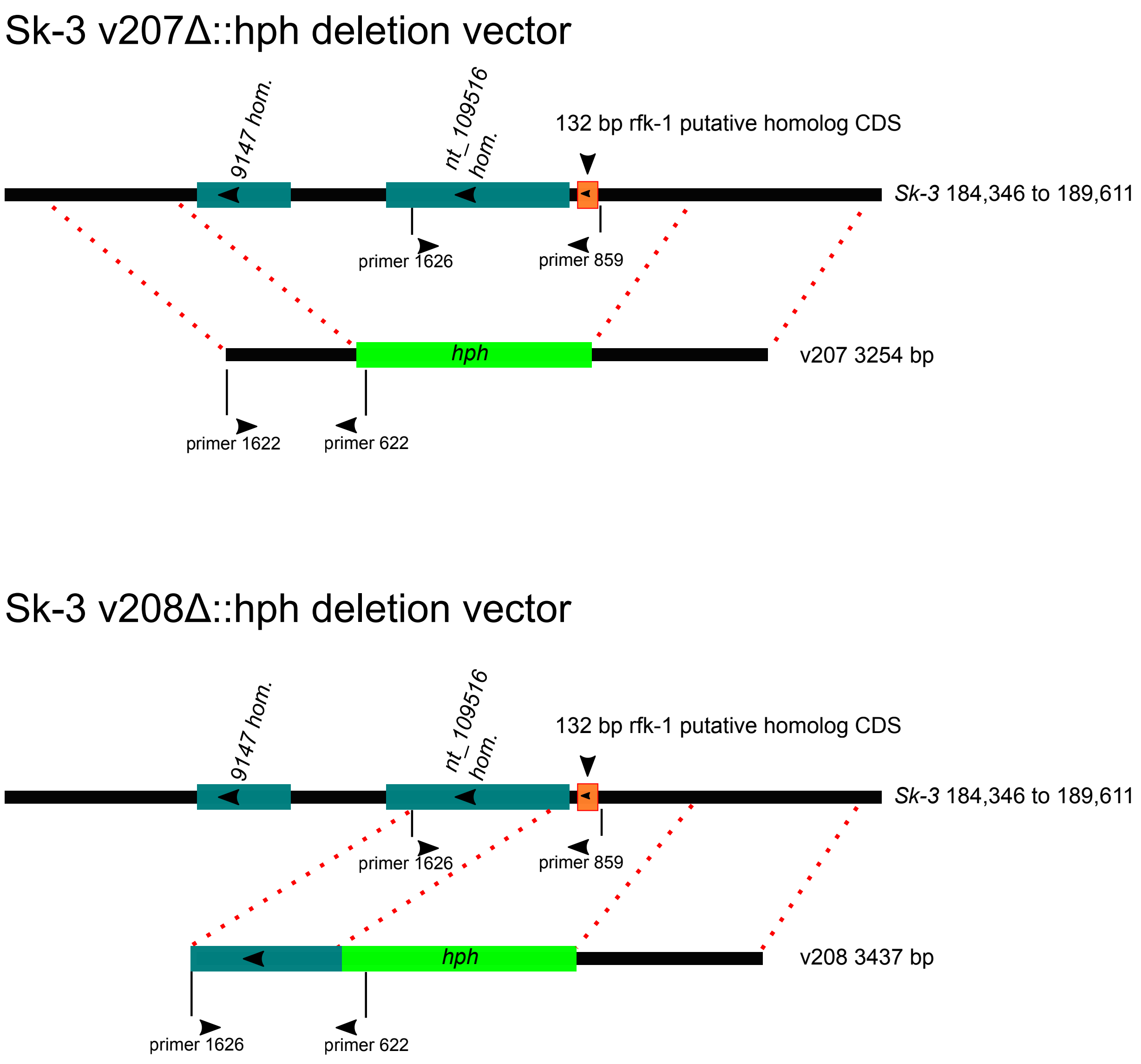


**Supplementary Figure 5**

Schematic of the two vectors used to delete the fragment in *Sk-3* which shows homology to the *rfk-1* killer gene in *Sk-2*.

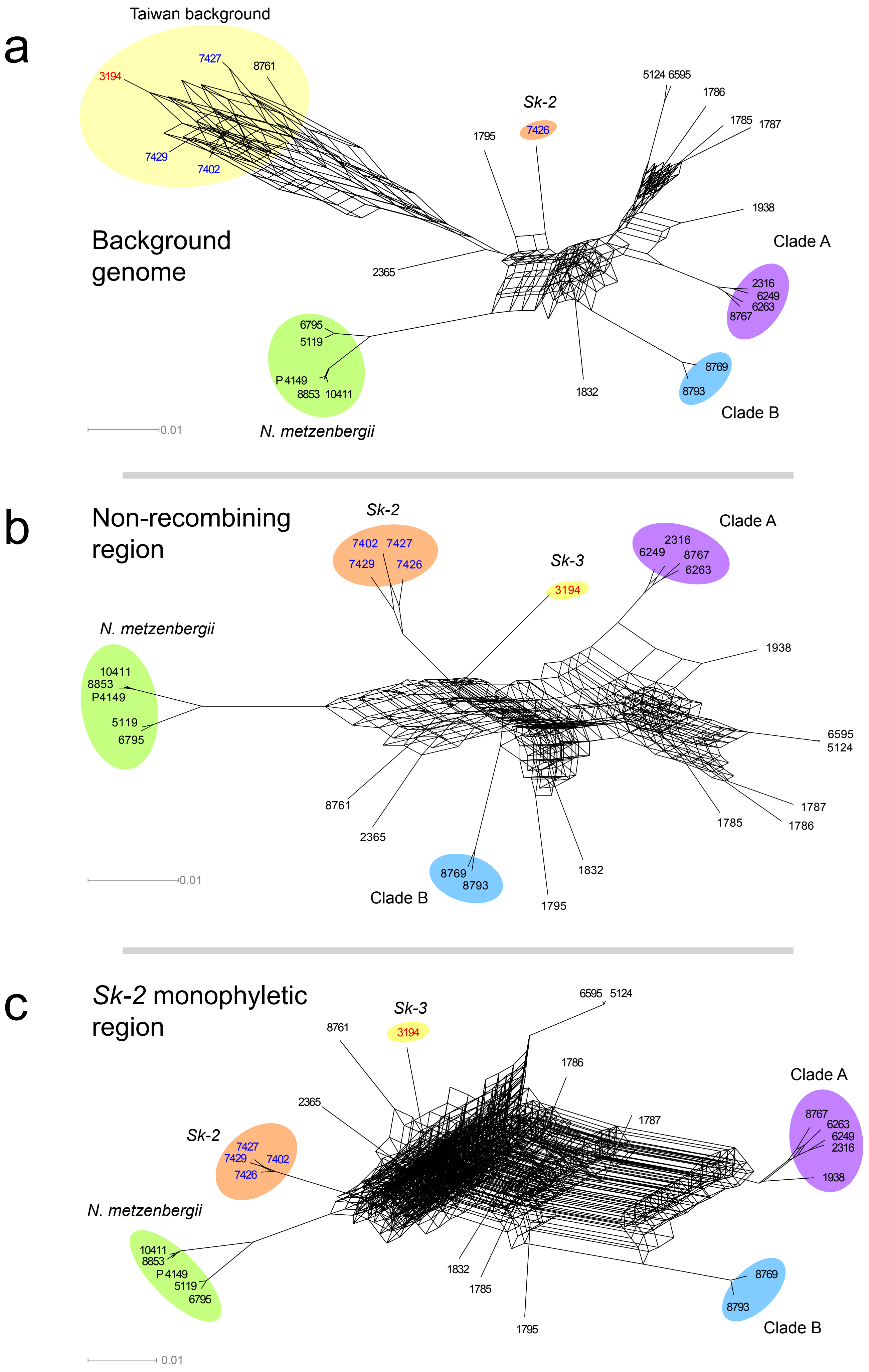
a
Taiwan background
Background genome
Sk-2
Clade A
Clade B
N. metzenbergii
3194
7427
8761
7429
7402
1795
7426
5124 6595
1786
1785
1787
1938
2365
2316
6249
6263
8767
6795
5119
P4149
8853
10411
1832
8769
8793
0.01
b
Non-recombining region
Sk-2
7402
7427
7429
7426
Sk-3
3194
Clade A
2316
6249
8767
6263
N. metzenbergii
10411
8853
P4149
5119
6795
1938
6595
5124
8761
2365
1787
1785
1786
1832
8769
8793
Clade B
1795
0.01
c
Sk-2 monophyletic region
Sk-3
3194
8761
6595
5124
1786
2365
1787
Clade A
8767
6263
6249
2316
1938
Sk-2
7427
7429
7402
7426
N. metzenbergii
10411
8853
P4149
5119
6795
1832
1785
1795
8769
8793
Clade B
0.01

**Supplementary Figure 6**

**a)** Network of *N. intermedia* and *N. metzenbergii* based on SNPs from all chromosomes, except chromosome 3. The network is constructed from phylogenetic trees generated using RAxML of each chromosome independently, the four spore killer strains clustering with the Taiwan strain 8761 are highlighted, showing the signal of the back-crossing into the Taiwan background, as are the ones grouping together with either of *N. intermedia* clades A and B, and of the outgroup, *N. metzenbergii*. **b)** Network based on SNPs in the nonrecombining region. The network is constructed using phylogenetic trees independently generated using RAxML from the five inversions in *Sk-2*. All *Sk-2* strains cluster together, but *Sk-3* is placed elsewhere in the network. **c)** Network based on SNPs from the regions where the four *Sk-2* strains show a signal of monophyly. In all networks, *Sk-2* strains are marked with red text, and the *Sk-3* strain is marked with blue text.

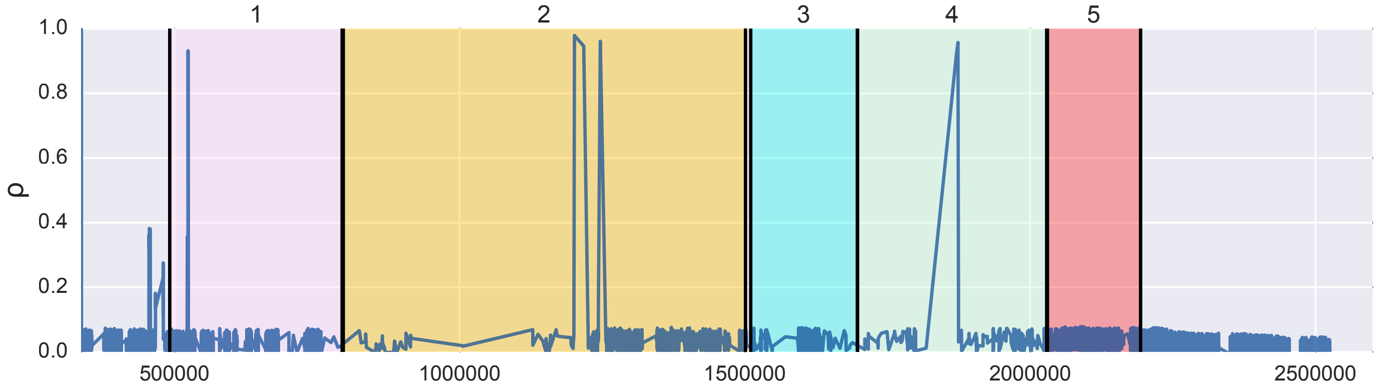


**Supplementary Figure 7**
Population scaled recombination rate (ρ) for the four *Sk-2* strains was estimated using LDhat plotted over the *Sk* region of chromosome 3. The five inversions are marked in color and numbered. There is little evidence for recombination between the *Sk-2* strains, besides four narrow peaks. They could potentially be due to gene conversion, but mutation or mapping error cannot be excluded.

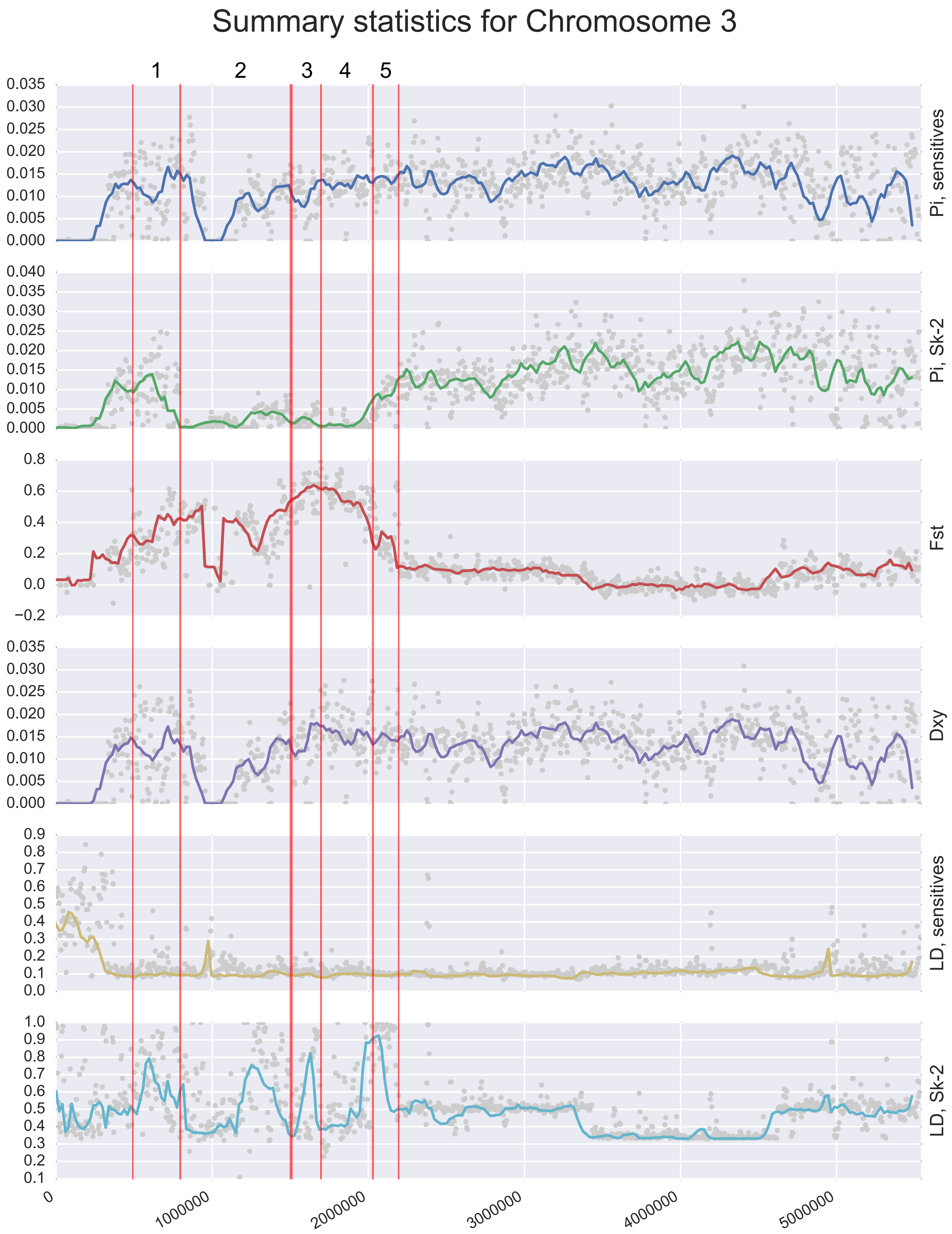


**Supplementary Figure 8**

Diversity, divergence and linkage disequilibrium plotted over chromosome 3. $F_{ST}$ and $D_{XY}$ is calculated by comparing *Sk-2* strains to sensitive *N. intermedia* strains. The non-recombining spore killer region is located between ~300 kbp and ~2.3 Mbp and inversion break-points are marked with red lines.

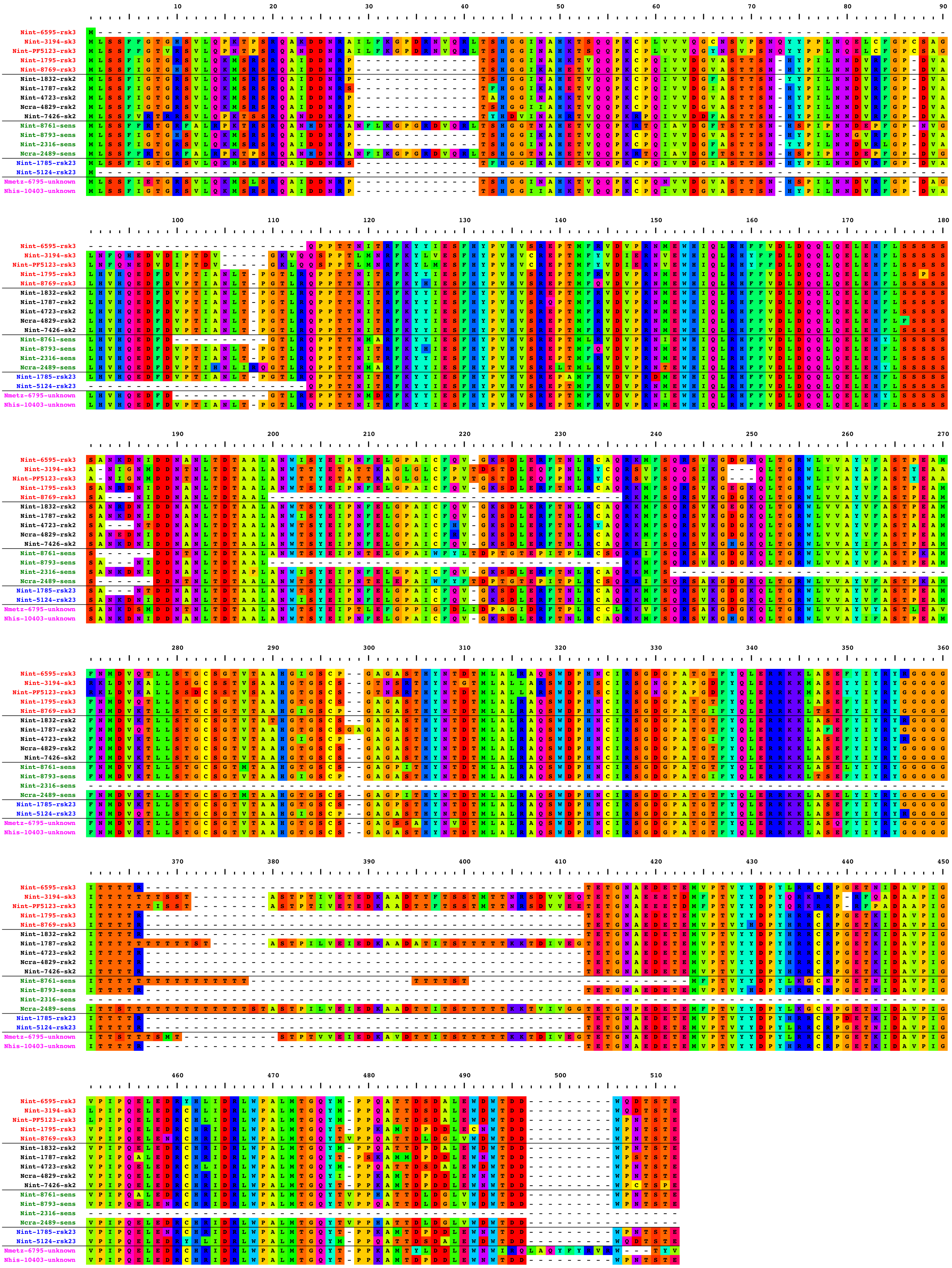

## Supplementary Figure 9

Amino acid alignment of *rsk*. Strain colors: resistance against *Sk-3*, red; resistance against *Sk-2*, black; resistance against both Sk-2 and Sk-3, blue; sensitive strains, green and unknown phenotype, magenta. No sites show fixed differences between any of the phenotypes.

**Supplementary Table 1: Types of data generated for *N. intermedia* strains**

| Strain [1] | Phenotype [2] | Location | Illumina | PacBio | BS-seq | ChIP-seq | RNA-seq |
|---|---|---|---|---|---|---|---|
| 1785 | *r(Sk-2) + r(Sk-3)* [3] | Papua New Guinea | X | | | | |
| 1786 | *r(Sk-3)* | Papua New Guinea | X | | | | |
| 1787 | *r(Sk-2)* | Papua New Guinea | X | | | | |
| 1795 | *r(Sk-3)* | Indonesia | X | | | | |
| 1832 | *r(Sk-2)* | Australia | X | | | | |
| 1938 | Sensitive | Papua New Guinea | X | | | | |
| 2316 | Sensitive | USA | X | | | | |
| 2365 | Sensitive | Hawaii | X | | | | |
| 3194 [4] | *Sk-3* | Papua New Guinea | X | X | X | X | X |
| 5124 | *r(Sk-2) + r(Sk-3)* [3] | Tahiti | X | | | | |
| 6249 | Sensitive | Haiti | X | | | | |
| 6263 | Sensitive | Ivory coast | X | | | | |
| 6595 | *r(Sk-3)* | Tahiti | X | | | | |
| 7401 [4, 5] | *Sk-2* | Brunei | | X | | | |
| 7402 [4, 5] | *Sk-2* | Brunei | X | | | | |
| 7426 | *Sk-2* | Sabah | X | X | X | X | X |
| 7427 [4] | *Sk-2* | Java | X | X | | | |
| 7429 [4] | *Sk-2* | Papua New Guinea | X | X | | | |
| 8761 | Sensitive | Taiwan | X | X | X | X | X |
| 8762 | Sensitive | Taiwan | | | X | | X |
| 8767 | *r(Sk-2)* | Indonesia | X | | | | |
| 8769 | *r(Sk-3)* | Indonesia | v | | | | |
| 8793 | Sensitive | Papua New Guinea | X | | | | |
| 8807 [6] | Sensitive | Indonesia | | X | | | |

[1] Strains are referred to by their accession number at the Fungal Genetics Stock Center, see http://www.fgsc.net

[2] *r(Sk-2)* indicates resistant to *Sk-2* and *r(Sk-3)* resistant to *Sk-3*.

[3] Strain 1785 and 5124 show partial resistance to both *Sk-2* and *Sk-3*.

[4] These strains have been backcrossed 1-3 times to the “Taiwan” *N. intermedia* tester strains (FGSC strains 8761 and 8762).

[5] 7401 and 7402 are the two mating types isolated from a single backcross to the “Taiwan” background.

[6] Strain 8807 was sequenced together with the other strains in this study, but has been released in an earlier study[1].

**Supplementary Table 2: Location and size of the *Sk* region in 7426 and 3194, and of inversions within it.**

| Strain | | Start coordinate [1] | End coordinate [1] | Size (bp) | *N. crassa* start [2] | *N. crassa* end [2] |
|---|---|---|---|---|---|---|
| ***Sk-2*, 7426** | | | | | | |
| Whole region | | 146,604 | 2,744,839 | 2,598,235 | 167,108 | 2,011,073 |
| Inversion | 1 | 146,604 | 505,422 | 358,818 | 167,222 | 503,812 |
| | 2 | 735,396 | 1,601,169 | 865,773 | 506,357 | 1,310,671 |
| | 3 | 1,621,721 | 2,016,643 | 394,922 | 1,318,300 | 1,486,497 |
| | 4 | 2,057,118 | 2,549,759 | 492,641 | 1,487,028 | 1,845,202 |
| | 5 | 2,551,222 | 2,733,837 | 182,615 | 1,846,558 | 2,010,416 |
| ***Sk-3*, 3194** | | | | | | |
| Whole region | | 218,817 | 3144,883 | 2,926,066 | 162,630 | 2,594,373 |
| Inversion | 1 | 220,519 | 291,562 | 71,043 | 167,437 | 241,256 |
| | 2 | 291,595 | 315,284 | 23,689 | 241,099 | 264,955 |
| | 3 | 318,455 | 327,952 | 9,497 | 273,483 | 282,880 |
| | 4 | 333,059 | 1,267,606 | 934,547 | 283,021 | 1,192,260 |
| | 5 | 1,370,044 | 1,627,213 | 257,169 | 1,192,579 | 1,359,520 |
| | 6 | 1,663,134 | 1,845,276 | 182,142 | 1,361,585 | 1,544,816 |
| | 7 | 1,922,683 | 2,895,612 | 972,929 | 1,548,552 | 2,594,215 |

[1] Start and end coordinates are the coordinates for the breakpoints in *Sk-2* strain 7426 and *Sk-3* strain 3194.

[2] *N. crassa* start and end are the coordinates for the breakpoints when aligning *Sk-2* and *Sk-3* to the *N. crassa* OR74 genome[2].

**Supplementary Table 3. The 20 most common repeat families as defined by total sequence length.**

| Sk-2, 7426 | | | | | |
|---|---|---|---|---|---|
| **Whole genome** | | | **Spore killer region** | | |
| **Type [1]** | **Counts** | **Total (bp)** | **Type [1]** | **Counts** | **Total (bp)** |
| ncra_Tad1_01 | 136 | 494796 | ncra_Tad1_01 | 21 | 99170 |
| ncra_LTR_40 | 42 | 353924 | ncra_LTR_48 | 15 | 82023 |
| ncra_LTR_49 | 78 | 335496 | ncra_LTR_40 | 12 | 75155 |
| ncra_LTR_48 | 69 | 333100 | ndisc_LTR_48 | 15 | 33476 |
| ncra_Gypsy_08 | 34 | 154516 | ntet_LTR_14 | 12 | 32515 |
| ndisc_LTR_48 | 47 | 148203 | ncra_Gypsy_01 | 11 | 27959 |
| ncra_LTR_44 | 27 | 144423 | ncra_LTR_44 | 5 | 26124 |
| ncra_Tad1_06 | 58 | 108439 | ncra_LTR_82 | 5 | 23795 |
| ncra_Gypsy_01 | 34 | 104891 | ncra_Tad1_06 | 8 | 22913 |
| ntet_LTR_27 | 64 | 103611 | ncra_LTR_86 | 7 | 22692 |
| ncra_LTR_86 | 50 | 95745 | ncra_Gypsy_08 | 5 | 21377 |
| ncra_Gypsy_10 | 16 | 90772 | ntet_LTR_27 | 9 | 19032 |
| ncra_LTR_111 | 173 | 87862 | ncra_LTR_42 | 7 | 17323 |
| ncra_LTR_82 | 17 | 79224 | ncra_Gypsy_10 | 2 | 16450 |
| ncra_RPTFAM_02 | 54 | 73894 | ncra_LTR_49 | 8 | 16026 |
| ncra_LTR_34 | 90 | 71248 | ntet_LTR_16 | 5 | 14275 |
| ncra_LTR_93 | 29 | 71184 | ncra_LTR_34 | 11 | 12468 |
| ncra_Gypsy_13 | 36 | 70232 | ndis_RPTFAM_105 | 13 | 12277 |
| ntet_LTR_16 | 36 | 69150 | ncra_RPTFAM_02 | 9 | 11973 |
| ncra_LTR_33 | 56 | 67843 | ncra_LTR_111 | 19 | 11426 |

| Sk-3, 3194 | | | | | |
|---|---|---|---|---|---|
| **Whole genome** | | | **Spore killer region** | | |
| **Type [1]** | **Counts** | **Total (bp)** | **Type [1]** | **Counts** | **Total (bp)** |
| ncra_Tad1_01 | 128 | 445762 | ncra_Tad1_01 | 23 | 99425 |
| ncra_Gypsy_08 | 49 | 254516 | ncra_LTR_40 | 7 | 56498 |
| ncra_LTR_48 | 88 | 243225 | ncra_Gypsy_01 | 10 | 34910 |
| ncra_LTR_40 | 22 | 223803 | ncra_LTR_86 | 14 | 31598 |
| ncra_Gypsy_01 | 57 | 200126 | ncra_LTR_48 | 7 | 27225 |
| ncra_LTR_86 | 80 | 169734 | ncra_LTR_05 | 10 | 24234 |
| ncra_LTR_49 | 51 | 143704 | ndisc_LTR_48 | 7 | 24108 |
| ntet_LTR_16 | 56 | 141634 | ncra_LTR_49 | 7 | 19828 |
| ndisc_LTR_48 | 35 | 123693 | ndisc_LTR_58 | 10 | 18402 |
| ncra_LTR_93 | 39 | 101865 | ncra_Tad1_06 | 9 | 16600 |
| ncra_LTR_05 | 40 | 100834 | ntet_LTR_14 | 5 | 15452 |
| ncra_Tad1_06 | 50 | 98002 | ncra_LTR_69 | 2 | 14975 |
| ncra_LTR_75 | 22 | 94813 | ncra_Gypsy_08 | 2 | 14352 |
| ncra_Gypsy_10 | 12 | 81946 | ncra_LTR_122 | 3 | 13929 |
| ntet_LTR_27 | 57 | 76058 | ncra_LTR_111 | 27 | 12176 |
| ncra_LTR_111 | 152 | 73566 | ncra_LTR_34 | 6 | 12095 |
| ncra_LTR_69 | 13 | 69297 | ndis_Gypsy_03 | 6 | 11166 |
| ncra_LTR_04 | 21 | 68337 | ncra_Gypsy_13 | 9 | 10696 |

| | | | | | |
|---|---|---|---|---|---|
| ncra_Gypsy_13 | 38 | 68187 | ncra_LTR_45 | 10 | 10509 |
| ncra_LTR_44 | 20 | 63517 | ndisc_nonLTR_61 | 9 | 10416 |

| **Sensitive, 8761** | | | | | |
|---|---|---|---|---|---|
| **Whole genome** | | | **Spore killer region** | | |
| **Type [1]** | **Counts** | **Total (bp)** | **Type [1]** | **Counts** | **Total (bp)** |
| ncra_Tad1_01 | 110 | 375887 | ntet_LTR_14 | 6 | 13030 |
| ncra_Gypsy_01 | 77 | 281007 | ncra_LTR_86 | 6 | 12332 |
| ncra_LTR_40 | 21 | 249242 | ncra_LTR_04 | 3 | 12190 |
| ncra_LTR_86 | 103 | 229409 | ncra_LTR_40 | 1 | 12149 |
| ncra_Gypsy_08 | 44 | 205203 | ncra_Gypsy_01 | 4 | 10466 |
| ncra_LTR_48 | 68 | 197506 | ncra_LTR_34 | 9 | 8216 |
| ncra_LTR_49 | 50 | 161543 | ncra_LTR_36 | 1 | 8061 |
| ncra_LTR_75 | 33 | 150945 | ncra_Tad1_01 | 2 | 7781 |
| ncra_LTR_05 | 30 | 93292 | ncra_LTR_69 | 1 | 7516 |
| ntet_LTR_16 | 41 | 86784 | ncra_LTR_48 | 2 | 7236 |
| ncra_LTR_93 | 33 | 84967 | ncra_LTR_75 | 2 | 7164 |
| ndisc_LTR_48 | 20 | 82389 | ndisc_LTR_44 | 3 | 6417 |
| ncra_LTR_34 | 91 | 75223 | ndis_Copia_01 | 1 | 5401 |
| ncra_LTR_33 | 55 | 73303 | ncra_RPTFAM_171 | 5 | 5177 |
| ncra_Tad1_06 | 42 | 72608 | GA-rich | 61 | 3618 |
| ncra_LTR_63 | 11 | 68558 | ncra_LTR_72 | 1 | 3545 |
| ncra_LTR_21 | 12 | 67224 | nsub_Gypsy_05 | 1 | 2901 |
| ncra_RPTFAM_02 | 47 | 64518 | ncra_Tad1_06 | 1 | 2330 |
| ncra_LTR_111 | 124 | 61505 | ncra_LTR_16 | 1 | 2311 |
| ntet_LTR_27 | 43 | 58414 | ncra_RPTFAM_10 | 9 | 2279 |

| ***N. crassa*** | | | | | |
|---|---|---|---|---|---|
| **Whole genome** | | | **Spore killer region** | | |
| **Type [1]** | **Counts** | **Total (bp)** | **Type [1]** | **Counts** | **Total (bp)** |
| ncra_Tad1_01 | 198 | 514976 | ncra_Tad1_01 | 33 | 97465 |
| ncra_Gypsy_01 | 95 | 249139 | ncra_Gypsy_01 | 9 | 29693 |
| ncra_LTR_86 | 90 | 224242 | ncra_LTR_86 | 8 | 21062 |
| ncra_RPTFAM_02 | 81 | 150143 | ncra_LTR_49 | 4 | 18418 |
| ncra_Gypsy_08 | 26 | 92483 | ncra_LTR_40 | 4 | 14829 |
| ncra_LTR_04 | 24 | 87427 | ncra_LTR_56 | 3 | 11470 |
| ncra_LTR_40 | 19 | 84761 | ncra_nonLTR_55 | 2 | 11334 |
| ncra_LTR_44 | 13 | 84511 | ncra_LTR_34 | 7 | 10939 |
| ncra_LTR_33 | 63 | 82717 | ncra_LTR_53 | 4 | 10556 |
| ncra_LTR_93 | 25 | 73040 | ncra_Gypsy_08 | 2 | 10251 |
| ncra_RPTFAM_16 | 32 | 70961 | ncra_RPTFAM_188 | 6 | 9390 |
| ncra_LTR_05 | 31 | 70784 | ncra_LTR_112 | 1 | 8604 |
| ncra_Gypsy_13 | 26 | 63481 | ncra_LTR_05 | 4 | 8533 |
| ncra_RPTFAM_05 | 40 | 60701 | ncra_LTR_62 | 1 | 7922 |
| GA-rich | 961 | 53916 | ncra_LTR_48 | 2 | 7763 |
| ncra_LTR_06 | 12 | 52298 | ncra_LTR_58 | 1 | 7566 |

| | | | | | |
|---|---|---|---|---|---|
| ncra_LTR_21 | 12 | 51976 | ncra_LTR_33 | 6 | 7525 |
| ncra_LTR_75 | 9 | 50191 | ncra_LTR_50 | 1 | 7325 |
| ncra_LTR_83 | 7 | 48929 | ncra_LTR_75 | 1 | 6922 |
| ncra_Gypsy_10 | 7 | 48237 | ncra_RPTFAM_16 | 4 | 6739 |

[1] Transposable family names are taken from the repeat library generated by Gioti et al. (2012)[3].

## Supplementary Table 4: Repeats enriched in Spore killer region

| | | Whole genome | | Spore killer region | | Enrichment | | Total number of repeats | | | |
|---|---|---|---|---|---|---|---|---|---|---|---|
| Strain | Repeat type [1] | Copies | Base pairs | Copies | Base pairs | Copies | Bases | Whole genome | Sk region | p-value | Bonferroni corrected significance |
| 3194 | ndisc_LTR_58 | 15 | 34473 | 10 | 18402 | 8.08 | 6.47 | 26157 | 2158 | 2.97E-08 | 5.44E-05 |
| 3194 | ncra_LTR_45 | 16 | 19125 | 10 | 10509 | 7.58 | 6.66 | 26157 | 2158 | 7.33E-08 | 5.44E-05 |
| 3194 | ncra_LTR_53 | 52 | 26534 | 17 | 7323 | 3.96 | 3.35 | 26157 | 2158 | 4.95E-07 | 5.44E-05 |
| 3194 | (TTA)n | 76 | 3737 | 18 | 914 | 2.87 | 2.96 | 26157 | 2158 | 3.64E-05 | 5.44E-05 |
| 3194 | (TAA)n | 106 | 5251 | 22 | 1105 | 2.52 | 2.55 | 26157 | 2158 | 4.75E-05 | 5.44E-05 |
| 7426 | (TTA)n | 94 | 4652 | 26 | 1393 | 3.58 | 3.87 | 26539 | 2053 | 7.26E-09 | 5.73E-05 |
| 7426 | ncra_LTR_45 | 29 | 40562 | 12 | 10616 | 5.35 | 3.38 | 26539 | 2053 | 6.80E-07 | 5.73E-05 |
| 7426 | ndisc_LTR_48 | 47 | 148203 | 15 | 33476 | 4.13 | 2.92 | 26539 | 2053 | 1.46E-06 | 5.73E-05 |
| 7426 | (TAA)n | 96 | 5121 | 22 | 1248 | 2.96 | 3.15 | 26539 | 2053 | 3.30E-06 | 5.73E-05 |
| 7426 | (TAT)n | 84 | 3773 | 20 | 999 | 3.08 | 3.42 | 26539 | 2053 | 4.88E-06 | 5.73E-05 |
| 7426 | ntet_LTR_14 | 34 | 65726 | 12 | 32515 | 4.56 | 6.40 | 26539 | 2053 | 4.98E-06 | 5.73E-05 |
| 7426 | (AAT)n | 74 | 3807 | 18 | 837 | 3.14 | 2.84 | 26539 | 2053 | 1.04E-05 | 5.73E-05 |
| 7426 | (ATAATT)n | 9 | 486 | 6 | 303 | 8.62 | 8.06 | 26539 | 2053 | 1.47E-05 | 5.73E-05 |
| 7426 | ncra_Gypsy_01 | 34 | 104891 | 11 | 27959 | 4.18 | 3.45 | 26539 | 2053 | 3.16E-05 | 5.73E-05 |
| 7426 | ncra_LTR_42 | 14 | 37796 | 7 | 17323 | 6.46 | 5.92 | 26539 | 2053 | 3.49E-05 | 5.73E-05 |
| 7426 | ntet_LTR_13 | 4 | 4145 | 4 | 4145 | 12.93 | 12.93 | 26539 | 2053 | 3.58E-05 | 5.73E-05 |
| 7426 | ncra_LTR_40 | 42 | 353924 | 12 | 75155 | 3.69 | 2.75 | 26539 | 2053 | 5.59E-05 | 5.73E-05 |
| 7426 | ncra_LTR_101 | 55 | 19677 | 14 | 5364 | 3.29 | 3.52 | 26539 | 2053 | 5.68E-05 | 5.73E-05 |
| *N. crassa* | ncra_Tad1_01 | 198 | 514976 | 33 | 97465 | 2.51 | 2.85 | 25908 | 1718 | 9.99E-07 | 6.47E-05 |
| *N. crassa* | ncra_LTR_34 | 15 | 37026 | 7 | 10939 | 7.04 | 4.46 | 25908 | 1718 | 2.25E-05 | 6.47E-05 |

[1] Transposable family names are taken from the repeat library generated by Gioti et al. (2012)[3].

## Supplementary Table 5: Bisulfite sequencing statistics

| Strain | Phenotype | Mating type | Sample name | Number of reads | Read length | Total data (bp) | Coverage |
|---|---|---|---|---|---|---|---|
| 3194 | *Sk-3* | mat A | SK3-1 | 17,662,450 | 126 | 4,450,937,400 | 106.7 |
| | | | SK3-2 | 16,514,315 | 126 | 4,161,607,380 | 99.8 |
| | | | SK3-3 | 19,851,533 | 126 | 5,002,586,316 | 120.0 |
| 7426 | *Sk-2* | mat A | SK2-1 | 18,622,707 | 126 | 4,692,922,164 | 111.2 |
| | | | SK2-2 | 20,000,164 | 126 | 5,040,041,328 | 119.4 |
| | | | SK2-3 | 20,167,691 | 126 | 5,082,258,132 | 120.4 |
| 8761 | Sensitive | mat A | SenA-1 | 19,217,691 | 126 | 4,842,858,132 | 118.1 |
| | | | SenA-2 | 18,868,363 | 126 | 4,754,827,476 | 116.0 |
| | | | SenA-3 | 20,112,790 | 126 | 5,068,423,080 | 123.6 |
| 8762 | Sensitive | mat a | Sena-1 | 19,759,188 | 126 | 4,979,315,376 | 121.4 |
| | | | Sena-2 | 18,538,934 | 126 | 4,671,811,368 | 113.9 |
| | | | Sena-3 | 20,112,790 | 126 | 5,068,423,080 | 123.6 |

## Supplementary Table 6: ChIP-seq sequencing statistics

| Strain | Phenotype | Data type | Sample name | Number of reads | Read length | Total data (bp) | Coverage |
|---|---|---|---|---|---|---|---|
| 8761 | Sensitive | H3 | senA-H3-1 | 2,391,286 | 126 | 602,604,072 | 14.7 |
| | | | senA-H3-2 | 3,176,333 | 126 | 800,435,916 | 19.5 |
| | | | senA-H3-3 | 3,287,804 | 126 | 828,526,608 | 20.2 |
| 8761 | Sensitive | input | senA-input-1 | 2,786,779 | 126 | 702,268,308 | 17.1 |
| | | | senA-input-2 | 6,091,738 | 126 | 1,535,117,976 | 37.4 |
| | | | senA-input-3 | 5,861,906 | 126 | 1,477,200,312 | 36.0 |
| 8761 | Sensitive | H3K27me | senA-K27-1 | 4,539,789 | 126 | 1,144,026,828 | 27.9 |
| | | | senA-K27-2 | 5,614,113 | 126 | 1,414,756,476 | 34.5 |
| | | | senA-K27-3 | 3,431,904 | 126 | 864,839,808 | 21.1 |
| 8761 | Sensitive | H3K4me2 | senA-K4-1 | 1,939,960 | 126 | 488,869,920 | 11.9 |
| | | | senA-K4-2 | 5,623,058 | 126 | 1,417,010,616 | 34.6 |
| | | | senA-K4-3 | 5,177,248 | 126 | 1,304,666,496 | 31.8 |
| 8761 | Sensitive | H3K9me3 | senA-K9-1 | 5,532,061 | 126 | 1,394,079,372 | 34.0 |
| | | | senA-K9-2 | 7,274,107 | 126 | 1,833,074,964 | 44.7 |
| | | | senA-K9-3 | 3,484,261 | 126 | 878,033,772 | 21.4 |
| 7426 | *Sk-2* | H3 | SK2-H3-1 | 3,153,238 | 126 | 794,615,976 | 18.8 |
| | | | SK2-H3-2 | 3,850,303 | 126 | 970,276,356 | 23.0 |
| | | | SK2-H3-3 | 2,216,851 | 126 | 558,646,452 | 13.2 |
| 7426 | *Sk-2* | input | SK2-input-1 | 4,550,749 | 126 | 1,146,788,748 | 27.2 |
| | | | SK2-input-2 | 3,891,064 | 126 | 980,548,128 | 23.2 |
| | | | SK2-input-3 | 5,008,086 | 126 | 1,262,037,672 | 29.9 |
| 7426 | *Sk-2* | H3K27me | SK2-K27-1 | 4,610,774 | 126 | 1,161,915,048 | 27.5 |
| | | | SK2-K27-2 | 3,255,844 | 126 | 820,472,688 | 19.4 |
| | | | SK2-K27-3 | 2,947,638 | 126 | 742,804,776 | 17.6 |
| 7426 | *Sk-2* | H3K4me2 | SK2-K4-1 | 3,688,828 | 126 | 929,584,656 | 22.0 |
| | | | SK2-K4-2 | 4,434,644 | 126 | 1,117,530,288 | 26.5 |
| | | | SK2-K4-3 | 3,407,310 | 126 | 858,642,120 | 20.3 |
| 7426 | *Sk-2* | H3K9me3 | SK2-K9-1 | 7,226,248 | 126 | 1,821,014,496 | 43.2 |
| | | | SK2-K9-2 | 4,527,742 | 126 | 1,140,990,984 | 27.0 |
| | | | SK2-K9-3 | 4,247,728 | 126 | 1,070,427,456 | 25.4 |
| 3194 | *Sk-3* | H3 | SK3-H3-1 | 3,112,293 | 126 | 784,297,836 | 18.8 |
| | | | SK3-H3-2 | 4,821,281 | 126 | 1,214,962,812 | 29.1 |
| | | | SK3-H3-3 | 3,100,894 | 126 | 781,425,288 | 18.7 |
| 3194 | *Sk-3* | input | SK3-input-1 | 5,029,166 | 126 | 1,267,349,832 | 30.4 |
| | | | SK3-input-2 | 6,036,475 | 126 | 1,521,191,700 | 36.5 |
| | | | SK3-input-3 | 4,302,365 | 126 | 1,084,195,980 | 26.0 |
| 3194 | *Sk-3* | H3K27me | SK3-K27-1 | 3,404,053 | 126 | 857,821,356 | 20.6 |
| | | | SK3-K27-2 | 4,273,854 | 126 | 1,077,011,208 | 25.8 |
| | | | SK3-K27-3 | 2,893,884 | 126 | 729,258,768 | 17.5 |

| Strain | Phenotype | Data type | Sample name | Number of reads | Read length | Total data (bp) | Coverage |
|---|---|---|---|---|---|---|---|
| 3194 | *Sk-3* | H3K4me2 | SK3-K4-1 | 3,936,471 | 126 | 991,990,692 | 23.8 |
| | | | SK3-K4-2 | 4,723,153 | 126 | 1,190,234,556 | 28.5 |
| | | | SK3-K4-3 | 3,076,918 | 126 | 775,383,336 | 18.6 |
| 3194 | *Sk-3* | H3K9me3 | SK3-K9-1 | 4,653,903 | 126 | 1,172,783,556 | 28.1 |
| | | | SK3-K9-2 | 5,651,398 | 126 | 1,424,152,296 | 34.2 |
| | | | SK3-K9-3 | 3,914,395 | 126 | 986,427,540 | 23.7 |

## Supplementary Table 7: RNA-seq sequencing statistics for crosses

| Strain | Phenotype | Sample name | Number of reads | Read length (bp) | Total data (bp) |
|---|---|---|---|---|---|
| 8761 x 8762 | Sens x sens | CrossA-1 | 52,471,010 | 126 | 13,222,694,520 |
| 8761 x 8762 | Sens x sens | CrossA-2 | 20,993,267 | 126 | 5,290,303,284 |
| 8761 x 8762 | Sens x sens | CrossA-3 | 28,147,157 | 126 | 7,093,083,564 |
| 7426 x 8762 | *Sk-2* x sens | CrossB-1 | 20,736,078 | 126 | 5,225,491,656 |
| 7426 x 8762 | *Sk-2* x sens | CrossB-2 | 22,255,907 | 126 | 5,608,488,564 |
| 7426 x 8762 | *Sk-2* x sens | CrossB-3 | 18,808,203 | 126 | 4,739,667,156 |
| 3194 x 8762 | *Sk-3* x sens | CrossC-1 | 26,837,792 | 126 | 6,763,123,584 |
| 3194 x 8762 | *Sk-3* x sens | CrossC-2 | 35,555,809 | 126 | 8,960,063,868 |
| 3194 x 8762 | *Sk-3* x sens | CrossC-3 | 36,769,552 | 126 | 9,265,927,104 |

## Supplementary Table 8: RNA-seq sequencing statistics for vegetative samples

| Strain | Phenotype | Mating type | Sample name | Number of reads | Read length (bp) | Total data (bp) | Coverage |
|---|---|---|---|---|---|---|---|
| 7426 | *Sk-2* | *mat A* | SK2-1 | 18,422,104 | 126 | 4,642,370,208 | 110.0 |
| 7426 | *Sk-2* | *mat A* | SK2-2 | 24,658,673 | 126 | 6,213,985,596 | 147.3 |
| 7426 | *Sk-2* | *mat A* | SK2-3 | 15,867,499 | 126 | 3,998,609,748 | 94.8 |
| 3194 | *Sk-3* | *mat A* | SK3-1 | 22,771,549 | 126 | 5,738,430,348 | 137.6 |
| 3194 | *Sk-3* | *mat A* | SK3-2 | 15,605,292 | 126 | 3,932,533,584 | 94.3 |
| 3194 | *Sk-3* | *mat A* | SK3-3 | 27,762,217 | 126 | 6,996,078,684 | 167.8 |
| 8761 | Sensitive | *mat A* | SenA-1 | 29,809,029 | 126 | 7,511,875,308 | 183.2 |
| 8761 | Sensitive | *mat A* | SenA-2 | 38,073,840 | 126 | 9,594,607,680 | 234.0 |
| 8761 | Sensitive | *mat A* | SenA-3 | 15,273,227 | 126 | 3,848,853,204 | 93.9 |
| 8762 | Sensitive | *mat a* | Sena-1 | 18,594,466 | 126 | 4,685,805,432 | 114.3 |
| 8762 | Sensitive | *mat a* | Sena-2 | 18,133,803 | 126 | 4,569,718,356 | 111.5 |
| 8762 | Sensitive | *mat a* | Sena-3 | 15,521,625 | 126 | 3,911,449,500 | 95.4 |

## Supplementary Table 9: Unique transcripts in the Spore killer region

| Strain | Phenotype | Gene | Annotation | Chromosome | Start coordinate | End coordinate | Protein size |
|---|---|---|---|---|---|---|---|
| 3194 | *Sk-3* | maker-3194pb_lg3-snap-gene-0.220-mRNA-1 | - | lg3 | 848,540 | 849,297 | 197 |
| 3194 | *Sk-3* | snap_masked-3194pb_lg3-processed-gene-1.380-mRNA-1 | - | lg3 | 1,054,612 | 1,055,785 | 108 |
| 3194 | *Sk-3* | maker-3194pb_lg3-snap-gene-1.156-mRNA-1 | - | lg3 | 1,236,639 | 1,237,847 | 110 |
| 3194 | *Sk-3* | maker-3194pb_lg3-snap-gene-1.160-mRNA-1 | - | lg3 | 1,267,791 | 1,268,487 | 193 |
| 7426 | *Sk-2* | maker-7426pb_lg3-est_gff_Cufflinks-gene-0.212-mRNA-1 | - | lg3 | 834,238 | 835,468 | 166 |
| 7426 | *Sk-2* | maker-7426pb_lg3-snap-gene-0.47-mRNA-1 | - | lg3 | 887,375 | 892,546 | 59 |
| 7426 | *Sk-2* | maker-7426pb_lg3-snap-gene-1.7-mRNA-1 | annexin A7-like | lg3 | 1,188,494 | 1,189,250 | 107 |
| 7426 | *Sk-2* | maker-7426pb_lg3-snap-gene-1.24-mRNA-1 | Peptidase_S8 | lg3 | 1,557,848 | 1,561,995 | 1028 |
| 7426 | *Sk-2* | maker-7426pb_lg3-snap-gene-1.30-mRNA-1 | - | lg3 | 1,648,309 | 1,649,385 | 60 |
| 7426 | *Sk-2* | maker-7426pb_lg3-est_gff_Cufflinks-gene-1.165-mRNA-1 | - | lg3 | 1,721,388 | 1,722,228 | 140 |
| 8761 | Sensitive | maker-8761pb_lg3.1-snap-gene-0.477-mRNA-1 | - | lg3 | 284,264 | 284,823 | 161 |
| 8761 | Sensitive | maker-8761pb_lg3.1-snap-gene-0.487-mRNA-1 | - | lg3 | 350,887 | 351,224 | 106 |
| 8761 | Sensitive | snap-8761pb_lg3.1-processed-gene-0.52-mRNA-1 | - | lg3 | 358,752 | 359,078 | 100 |
| 8761 | Sensitive | maker-8761pb_lg3.1-snap-gene-0.500-mRNA-1 | - | lg3 | 483,926 | 484,259 | 97 |
| 8761 | Sensitive | snap-8761pb_lg3.1-processed-gene-0.70-mRNA-1 | Procollagen type XV | lg3 | 540,460 | 540,852 | 74 |
| *N. crassa* | Sensitive | NCU09164T0 | - | lg3 | 224,366 | 225,339 | 105 |
| *N. crassa* | Sensitive | NCU10629T0 | - | lg3 | 328,150 | 328,634 | 133 |
| *N. crassa* | Sensitive | NCU05657T0 | - | lg3 | 388,933 | 390,336 | 264 |
| *N. crassa* | Sensitive | NCU05662T0 | - | lg3 | 401,171 | 401,661 | 84 |

| Strain | Phenotype | Gene | Annotation | Chromosome | Start coordinate | End coordinate | Protein size |
|---|---|---|---|---|---|---|---|
| *N. crassa* | Sensitive | NCU05742T0 | - | lg3 | 813,119 | 813,977 | 103 |
| *N. crassa* | Sensitive | NCU16630T0 | Has domain(s) with predicted RNA binding, RNA-directed DNA polymerase activity, nucleic acid binding activity and role in DNA integration, RNA-dependent DNA replication | lg3 | 1,336,781 | 1,337,245 | 155 |
| *N. crassa* | Sensitive | NCU06427T0 | - | lg3 | 1,377,695 | 1,378,620 | 128 |
| *N. crassa* | Sensitive | NCU06480T0 | - | lg3 | 1,554,709 | 1,555,852 | 213 |
| *N. crassa* | Sensitive | NCU06234T0 | - | lg3 | 1,998,310 | 1,999,669 | 114 |
| *N. crassa* | Sensitive | NCU10274T0 | - | lg3 | 2,126,140 | 2,127,451 | 156 |

**Supplementary Table 10: Genes in the *Sk* region in *Sk-2* and *Sk-3* but not sensitive strains**

| Ortholog group | ***Sk-2* gene** | | | | ***Sk-3* gene** | | | | Annotation |
|---|---|---|---|---|---|---|---|---|---|
| | Name | Start coordinate | End coordinate | Protein size | Name | Start coordinate | End coordinate | Protein size | |
| g8661 [1] | snap-7426pb_lg3-processed-gene-1.183-mRNA-1 | 1166491 | 1171390 | 1359 | maker-3194pb_lg3-snap-gene-0.141-mRNA-1 | 553888 | 558728 | 1298 | Unknown function. |
| g8662 [1] | maker-7426pb_lg3-snap-gene-1.61-mRNA-1 | 1160146 | 1165796 | 1249 | maker-3194pb_lg3-snap-gene-0.215-mRNA-1 | 548640 | 553430 | 1316 | Putative DNA transposon similar to Sly1-1. |
| g8741 | snap-7426pb_lg3-processed-gene-0.248-mRNA-1 | 206934 | 207542 | 202 | snap-3194pb_lg3-processed-gene-1.241-mRNA-1 | 1160484 | 1161293 | 269 | Unknown function. |

[1] The genes from ortholog group g8661 and g8662 are found next to each other in both the *Sk-2* and *Sk-3* genomes, but at different locations within the inverted regions in each genome. Potentially together both genes form a DNA transposon similar to *Sly1-1*.

## Supplementary Table 11: Illumina HiSeq sequencing and assembly statistics

| Species | Phenotype | Location | Strain | Contigs | Assembly size (bp) | N90 | N80 | N50 | N20 | Largest contig | Number of reads | Read length | Total data (Gbp) | Coverage |
|---|---|---|---|---|---|---|---|---|---|---|---|---|---|---|
| *N. intermedia* | *r(Sk-3)* | Papua New Guinea | 1785 | 1427 | 41,090,782 | 22,979 | 36,251 | 79,388 | 139,516 | 385,06 | 7,243,053 | 124 | 1.80 | 43.7 |
| *N. intermedia* | *r(Sk-3)* | Papua New Guinea | 1786 | 2006 | 41,240,449 | 21,417 | 35,582 | 74,694 | 141,476 | 299,644 | 7,986,704 | 124 | 1.98 | 48.0 |
| *N. intermedia* | *r(Sk-2)* | Papua New Guinea | 1787 | 2310 | 41,013,218 | 17,942 | 28,138 | 59,684 | 112,607 | 302,963 | 7,552,672 | 124 | 1.87 | 45.7 |
| *N. intermedia* | *r(Sk-3)* | Indonesia | 1795 | 1158 | 40,300,591 | 30,2 | 52,525 | 100,035 | 179,165 | 458,907 | 9,630,836 | 124 | 2.39 | 59.3 |
| *N. intermedia* | *r(Sk-2)* | Australia | 1832 | 1461 | 40,871,442 | 25,71 | 39,38 | 84,475 | 152,06 | 381,938 | 7,691,056 | 124 | 1.91 | 46.7 |
| *N. intermedia* | Sensitive | Papua New Guinea | 1938 | 1495 | 39,275,068 | 23,946 | 42,525 | 85,994 | 166,362 | 580,129 | 10,554,317 | 124 | 2.62 | 66.6 |
| *N. intermedia* | Sensitive | USA | 2316 | 1640 | 38,668,777 | 22,506 | 37,398 | 75,639 | 145,963 | 333,43 | 13,342,846 | 124 | 3.31 | 85.6 |
| *N. intermedia* | Sensitive | Hawaii | 2365 | 1566 | 41,437,638 | 31,559 | 58,355 | 109,578 | 221,442 | 495,884 | 12,762,957 | 124 | 3.17 | 76.4 |
| *N. intermedia* | *Sk-3* | Papua New Guinea | 3194 | 1284 | 41,405,991 | 30,312 | 46,192 | 93,356 | 172,444 | 412,602 | 9,595,575 | 124 | 2.38 | 57.5 |
| *N. intermedia* | Unknown | New Zealand | 5119 | 2264 | 42,735,677 | 22,091 | 39,298 | 86,059 | 160,525 | 368,397 | 11,135,305 | 124 | 2.76 | 64.6 |
| *N. intermedia* | *r(Sk-2) + r(Sk-3)* | Tahiti | 5124 | 4066 | 43,829,016 | 19,411 | 34,816 | 71,135 | 137,27 | 422,048 | 10,623,131 | 124 | 2.63 | 60.1 |
| *N. intermedia* | Sensitive | Haiti | 6249 | 1579 | 38,828,937 | 18,734 | 29,08 | 64,597 | 122,293 | 273,718 | 10,569,810 | 124 | 2.62 | 67.5 |
| *N. intermedia* | Sensitive | Ivory coast | 6263 | 1570 | 38,666,853 | 21,835 | 34,269 | 71,773 | 138,977 | 458,262 | 10,157,083 | 124 | 2.52 | 65.1 |
| *N. intermedia* | *r(Sk-3)* | Tahiti | 6595 | 2704 | 43,159,925 | 21,518 | 35,593 | 77,075 | 127,477 | 322,32 | 9,473,377 | 124 | 2.35 | 54.4 |
| *N. intermedia* | Unknown | New Zealand | 6795 | 2337 | 42,540,341 | 21,601 | 34,31 | 75,613 | 133,21 | 362,532 | 9,022,418 | 124 | 2.24 | 52.6 |
| *N. intermedia* | *Sk-2* | Brunei | 7402 | 1293 | 42,076,821 | 25,495 | 40,122 | 90,389 | 165,648 | 309,97 | 9,342,724 | 124 | 2.32 | 55.1 |
| *N. intermedia* | *Sk-2* | Sabah | 7426 | 1757 | 41,922,822 | 24,97 | 42,414 | 89,659 | 163,579 | 388,017 | 9,218,243 | 124 | 2.29 | 54.5 |
| *N. intermedia* | *Sk-2* | Java | 7427 | 1709 | 42,477,818 | 26,412 | 45,112 | 94,222 | 166,322 | 334,189 | 9,547,705 | 124 | 2.37 | 55.7 |
| *N. intermedia* | *Sk-2* | Papua New Guinea | 7429 | 1357 | 41,087,187 | 29,068 | 48,296 | 90,077 | 156,631 | 318,967 | 9,373,713 | 124 | 2.32 | 56.6 |
| *N. intermedia* | Sensitive | Taiwan | 8761 | 1762 | 41,071,115 | 23,212 | 37,929 | 75,851 | 147,727 | 326,559 | 9,459,755 | 124 | 2.35 | 57.1 |
| *N. intermedia* | *r(Sk-2)* | Indonesia | 8767 | 1676 | 37,775,537 | 15,552 | 24,292 | 52,492 | 99,573 | 220,91 | 6,921,303 | 124 | 1.72 | 45.4 |
| *N. intermedia* | *r(Sk-3)* | Indonesia | 8769 | 1194 | 39,799,185 | 23,381 | 37,153 | 78,057 | 160,447 | 440,184 | 9,175,022 | 124 | 2.28 | 57.2 |
| *N. intermedia* | Sensitive | Papua New Guinea | 8793 | 1147 | 39,486,933 | 21,7 | 34,444 | 70,635 | 132,168 | 348,704 | 9,462,259 | 124 | 2.35 | 59.4 |
| *N. intermedia* | Unknown | Mexico | P4149 | 4116 | 44,075,354 | 15,375 | 24,436 | 50,905 | 95,065 | 279,828 | 10,211,750 | 124 | 2.53 | 57.5 |
| *N. metzenbergii* | Unknown | Mexico | 8853 | 3291 | 43,176,579 | 16,611 | 25,566 | 57,213 | 100,504 | 273,505 | 9,143,136 | 124 | 2.27 | 52.5 |

| Species | Phenotype | Location | Strain | Contigs | Assembly size (bp) | N90 | N80 | N50 | N20 | Largest contig | Number of reads | Read length | Total data (Gbp) | Coverage |
|---|---|---|---|---|---|---|---|---|---|---|---|---|---|---|
| *N. metzenbergii* | Unknown | Mexico | 10411 | 3540 | 43,486,000 | 16,788 | 27,102 | 55,515 | 105,517 | 262,589 | 10,229,242 | 124 | 2.54 | 58.3 |
| *N. crassa* | *r(Sk-2)* | Malaya | 4723 | 1621 | 43,069,651 | 35,89 | 58,892 | 136,267 | 250,436 | 598,204 | 10,359,829 | 124 | 2.57 | 59.7 |
| *N. crassa* | *r(Sk-2)* | Ivory coast | 4829 | 1601 | 42,013,408 | 27,506 | 45,162 | 102,13 | 200,119 | 439,843 | 11,185,181 | 124 | 2.77 | 66.0 |
| *N. crassa* | Unknown | India | 8863 | 1279 | 40,248,786 | 27,037 | 41,9 | 86,864 | 166,428 | 349,317 | 9,596,092 | 124 | 2.38 | 59.1 |

**Supplementary Table 12: Age estimates of the non-recombining region of the *Sk-2* spore killers**

| Strain | Ds | Low age estimate (years) [1] | High age estimate (years) [2] |
|---|---|---|---|
| 7402 | 0.00512 | 256,411 | 430,081 |
| 7426 | 0.00505 | 252,794 | 424,014 |
| 7427 | 0.00510 | 255,472 | 428,505 |
| 7429 | 0.00510 | 255,370 | 428,334 |
| Mean | 0.00509 | 255,012 | 427,734 |

[1] Calculated using mutation rate of 9.98e-9 synonymous substitutions per site per year.

[2] Calculated using mutation rate of 5.95e-9 synonymous substitutions per site per year.

Mutation rates have been taken from Kasuga et al. (2002)[4].

**Table 13: Nucleotide diversity and divergence of two *N. intermedia* sensitive strains**

| ***N. intermedia* sensitive [1]** | $\pi_{total}$ | $\pi_N$ | $\pi_S$ | $\pi_{N/}\ \pi_S$ | $P_N$ | $P_S$ | $D_N$ | $D_S$ | $P_N$ / $P_S$ | $D_N$ / $D_S$ | NI |
|---|---|---|---|---|---|---|---|---|---|---|---|
| Total | 0.014 | 0.0076 | 0.040 | 0.191 | 52135 | 131114 | 63279 | 145860 | 0.40 | 0.43 | 0.92 |
| Chr1 | 0.013 | 0.0063 | 0.033 | 0.191 | 11040 | 27292 | 15037 | 33353 | 0.40 | 0.45 | 0.90 |
| Chr2 | 0.012 | 0.0075 | 0.038 | 0.194 | 5594 | 13658 | 6514 | 15853 | 0.41 | 0.41 | 1.00 |
| Chr3 [2] | 0.016 | 0.0077 | 0.038 | 0.201 | 4162 | 9315 | 5499 | 12668 | 0.45 | 0.43 | 1.03 |
| Chr4 | 0.014 | 0.0081 | 0.044 | 0.183 | 8384 | 22627 | 10157 | 22678 | 0.37 | 0.45 | 0.83 |
| Chr5 | 0.017 | 0.0071 | 0.040 | 0.179 | 7914 | 20999 | 8908 | 21767 | 0.38 | 0.41 | 0.92 |
| Chr6 | 0.013 | 0.0088 | 0.047 | 0.187 | 6325 | 16314 | 7101 | 16583 | 0.39 | 0.43 | 0.91 |
| Chr7 | 0.012 | 0.0092 | 0.045 | 0.206 | 6851 | 16456 | 7741 | 17170 | 0.42 | 0.45 | 0.92 |
| *Sk* reg. | 0.014 | 0.0075 | 0.037 | 0.202 | 1865 | 4453 | 2322 | 5788 | 0.42 | 0.40 | 1.04 |
| Bootstrap mean (95%) [3] | / | 0.0076 (0.0065, 0.0086) | 0.04 (0.037, 0.043) | 0.191 (0.16, 0.21) | 1990 (1646, 2284) | 4955 (4449, 5472) | 2381 (2113, 2678) | 5467 (5135, 5905) | 0.40 (0.35, 0.45) | 0.44 (0.39, 0.48) | 1.09 (0.96, 1.25) |

[1] Two haplotypes were selected for calculation of diversity and divergence.
[2] Chromosome 3 with Spore killer region excluded.
[3] Bootstrap confidence intervals were calculated by randomly picking the same number of genes as the number of genes in the Spore killer region. For bootstrap in total number of polymorphism and divergence, we also rescaled the number by the ratio of the total bootstrapped gene length and the length of the Spore killer region.

**Supplementary Table 14: Nucleotide diversity and divergence of nine *N. intermedia* sensitive strains**

| ***N. intermedia* sensitive [1]** | $\pi_{total}$ | $\pi_N$ | $\pi_S$ | $\pi_{N/}\ \pi_S$ | $P_N$ | $P_S$ | $D_N$ | $D_S$ | NI |
|---|---|---|---|---|---|---|---|---|---|
| Total | 0.014 | 0.0076 | 0.040 | 0.191 | 162822 | 418779 | 34189 | 68886 | 0.78 |
| Chr 1 | 0.013 | 0.0063 | 0.033 | 0.191 | 35709 | 87856 | 9190 | 17787 | 0.79 |
| Chr 2 | 0.012 | 0.0075 | 0.038 | 0.194 | 17093 | 44453 | 3435 | 7239 | 0.81 |
| Chr 3 [2] | 0.016 | 0.0077 | 0.038 | 0.201 | 13870 | 35545 | 2753 | 5467 | 0.77 |
| Chr 4 | 0.014 | 0.0081 | 0.044 | 0.183 | 26533 | 69961 | 4941 | 10097 | 0.78 |
| Chr 5 | 0.017 | 0.0071 | 0.040 | 0.179 | 25320 | 67771 | 4658 | 9972 | 0.80 |
| Chr 6 | 0.013 | 0.0088 | 0.047 | 0.187 | 18795 | 48628 | 3866 | 7712 | 0.77 |
| Chr 7 | 0.012 | 0.0092 | 0.045 | 0.206 | 19943 | 49764 | 4107 | 7834 | 0.76 |
| *Sk* region | 0.014 | 0.0075 | 0.037 | 0.202 | 5559 | 14801 | 1239 | 2778 | 0.84 |

[1] Nine haplotypes were selected for calculation of diversity and divergence
[2] Chromosome 3 with Spore killer region excluded

## Supplementary references


1. Sun, Y., Svedberg, J., Hiltunen, M., Corcoran, P. & Johannesson, H. Large-scale suppression of recombination predates genomic rearrangements in *Neurospora tetrasperma*. Nature Communications 8, 1140 (2017).
2. Galagan, J.E., Calvo, S.E., Borkovich, K.A., Selker, E.U., Read, N.D., Jaffe, D., FitzHugh, W., Ma, L.-J., Smirnov, S., Purcell, S., et al. (2003). The genome sequence of the filamentous fungus Neurospora crassa. Nature 422, 859–868.
3. Gioti, A., Mushegian, A. A., Strandberg, R., Stajich, J. E. & Johannesson, H. Unidirectional Evolutionary Transitions in Fungal Mating Systems and the Role of Transposable Elements. Mol Biol Evol 29, 3215–3226 (2012).
4. Kasuga, T., White, T. J. & Taylor, J. W. Estimation of Nucleotide Substitution Rates in Eurotiomycete Fungi. Mol Biol Evol 19, 2318–2324 (2002).
